# Macropinocytosis of Amyloid Precursor Protein Is Regulated by the Recruitment and Activity of Fe65, Arf6 and Rho GTPases

**DOI:** 10.3390/cells15151366

**Published:** 2026-07-29

**Authors:** Jordan M. Krupa, Manoj Reddy Medapati, Abdul M. Naqvi, Ryan D. Hallam, Adrianna R. Tsang, Claudia Seah, Shawn N. Whitehead, Stephen H. Pasternak

**Affiliations:** 1 Neuroscience Program, Schulich School of Medicine and Dentistry, University of Western Ontario, London, ON N6A 5B7, Canada; jkrupa2@uwo.ca (J.M.K.); anaqvi22@uwo.ca (A.M.N.); shawn.whitehead@uwo.ca (S.N.W.); 2 J. Allyn Taylor Centre for Cell Biology, Molecular Medicine Research Group, Robarts Research Institute, 1151 Richmond St, London, ON N6A 5B8, Canada; mmedapat@uwo.ca (M.R.M.); rhallam3@uwo.ca (R.D.H.); atsang57@uwo.ca (A.R.T.); cseah@uwo.ca (C.S.); 3 Department of Physiology and Pharmacology, Schulich School of Medicine, University of Western Ontario, London, ON N6A 5B7, Canada; 4 Department of Anatomy and Cell Biology, Schulich School of Medicine, University of Western Ontario, London, ON N6A 5B7, Canada; 5 Department of Clinical Neurological Sciences, Schulich School of Medicine, University of Western Ontario, London, ON N6A 5B7, Canada

**Keywords:** Alzheimer’s disease, amyloid precursor protein, macropinocytosis, lysosome, amyloid beta, Rho GTPase, endocytosis

## Abstract

**Highlights:**

**What are the main findings?**

- Fe65, Arf6 and the Rho GTPases Rac1, Cdc42, and RhoA are recruited to APP during its macropinocytosis to lysosomes.
- Mutation of the APP ‘YENPTY’ sequence, which mediates Fe65 binding, and inhibition of GTPase activity prevents the recruitment of GTPases to APP and its macropinocytosis to lysosomes.

**What are the implications of the main findings?**

- A network of regulatory proteins including Fe65, Arf6, Rac1, Cdc42 and RhoA regulate the macropinocytosis of APP.
- Targeting the macropinocytosis of APP through these regulatory proteins could be a novel strategy to reduce the production of amyloid-beta in Alzheimer’s disease.

**Abstract:**

Alzheimer’s disease (AD) is a progressive neurodegenerative disorder characterized by the buildup of aggregated amyloid-beta (Aβ) peptides. We previously demonstrated that Aβ is produced from APP following its lysosomal internalization via macropinocytosis. However, the regulation of APP macropinocytosis in neuronal cells remained uncharacterized. Arf6 and the Rho GTPases Rac1, Cdc42 and RhoA are known to regulate macropinocytosis in response to signaling at the cell surface. Fe65, an adaptor protein known to interact with APP, may link APP to these regulatory elements. We hypothesized that APP binding/crosslinking recruits Fe65, which recruits/activates Arf6 and then Rac1, Cdc42, and RhoA, driving APP macropinocytosis. We found that antibody-mediated binding/crosslinking APP resulted in the transient recruitment of Fe65 and Arf6 to APP within 30 s of APP binding/crosslinking. Rac1, Cdc42, and RhoA were also recruited at 30 s, but remained recruited through 2 min. The mutation of the APP ‘YENPTY’ sequence and Arf6 inhibition by NAV-2729 prevented the recruitment of Rac1, Cdc42, and RhoA. Together, these observations are the first to demonstrate that a network of regulatory proteins is recruited to bound/crosslinked APP and regulates its macropinocytosis. Targeting these regulatory proteins to modulate APP trafficking to the lysosome could be a therapeutic strategy to reduce Aβ production in AD.

## 1. Introduction

Alzheimer’s disease (AD) is a progressive neurodegenerative disease that affects approximately 43.8 million people worldwide, a number that is expected to significantly increase to 152 million by 2050 [1]. It is characterized on a cellular level by the formation of amyloid plaques containing aggregated amyloid-β (Aβ) peptides, and neurofibrillary tangles composed of aggregated tau protein [2,3]. Both Aβ peptides and soluble Aβ oligomers have been found to be toxic to neuronal synapses and dendritic spines, where synaptic loss is strongly correlated with impaired cognition in AD [4]. Moreover, Aβ can seed tau pathology, which tracks more closely with clinical disease progression [5].

The Aβ peptide is produced by the sequential enzymatic cleavage of the type-I transmembrane protein amyloid precursor protein (APP) by β-secretase (BACE) and γ-secretase [2,3]. Evidence suggests the lysosome is an important location for the amyloidogenic processing of APP. Intracellular Aβ colocalizes with autophagosomes and lysosomes [6,7], and lysosomal membranes are enriched in APP, APP β-C-terminal fragment (βCTF), and γ-secretase sub-units [8,9,10]. The lysosomal pH is also optimal for γ-secretase activity [11], and the inhibition of γ-secretase results in the accumulation of β-secretase cleaved amyloidogenic APP fragments in lysosomes [12]. The majority of Aβ is produced from APP after it is internalized from the plasma membrane, and therefore, examining the endocytosis of APP is critical to understanding the production of Aβ.

We previously identified a novel APP trafficking mechanism, in which APP is trafficked rapidly and directly from the plasma membrane to lysosomes within minutes, bypassing early and late endosomes [13]. We identified the underlying process as macropinocytosis [14], an actin-dependent endocytic mechanism that begins with local signaling to initiate membrane ruffling, which extends to form a macropinocytic cup, engulfing the extracellular fluid and/or membrane cargo [15]. Macropinocytic cups then collapse on the plasma membrane, forming macropinosomes, which rapidly fuse with lysosomes near the plasma membrane, delivering extracellular material, plasma membrane, and membrane proteins to the lysosome [16]. Using electron microscopy, we previously demonstrated APP within membrane ruffles at the plasma membrane, within macropinosomes, and in macropinosomes fusing with lysosomes [14]. Furthermore, APP colocalized at the membrane with CTBp1/BARS [14], which has been demonstrated to localize to macropinocytic cups, where it functions as part of a motor complex for macropinosome membrane fission [17]. Further, we also demonstrated that this process was actin-dependent by observing the reduced internalization of APP to lysosomes in response to the inhibition of actin polymerization using latrunculin B [14].

In our prior work investigating the macropinocytosis of APP, we utilized the binding and/or crosslinking of APP by fluorescently tagged antibodies to label and visualize cell surface APP. We have also previously provided evidence that the antibody binding/crosslinking of cell surface APP stimulates macropinocytosis. In our previous study, antibody-bound/crosslinked APP was rapidly internalized directly to lysosomes within 15 min, while unbound/uncrosslinked FlAsH-labeled APP was internalized primarily to early endosomes [14]. This method of stimulating APP macropinocytosis may relate to its physiological function, as APP has long been considered a cell surface receptor [18].

Many ligands have been demonstrated to bind to the extracellular portion of APP, including reelin, nerve growth factor (NGF), semaphorin-3a, and Aβ [19]. Within the extracellular portion of APP is a growth factor-like domain (GLFD), containing sites for copper binding, disulfide bridge formation and heparin binding domains [20]. It is worth noting that macropinocytosis can be stimulated through the binding of growth factors to receptors and the dimerization of these receptors [21]. All three of these domains play a role in the formation of APP homodimers, and the formation of homodimers has been shown to increase the production of Aβ [20,22,23]. The antibody-mediated binding and/or crosslinking of APP has also been shown to increase APP internalization and Aβ production [14,24]. Both macropinocytosis and APP have been shown to be upregulated in neurites and play important roles in neuronal growth cones and axonal guidance [25,26,27]. While a clear physiological function of APP–ligand binding has not yet been established, the antibody-mediated binding/crosslinking of APP currently serves as a model of the stimulation of APP macropinocytosis.

Many questions remain regarding the cellular mechanisms involved in APP macropinocytosis, particularly in neurons and neuronal cells. Several small GTPases have been demonstrated to regulate macropinocytosis in non-neuronal cells [28], including the regulatory GTPase Arf6, which is involved in its initiation [28,29]. The knockdown or dominant-negative mutations in Arf6 has been demonstrated to reduce the amount of APP located in lysosomes and Aβ production [14]. In brain samples from human AD patients, increased Arf6 expression has been demonstrated in hippocampal regions, suggesting that this GTPase may be important in pathological Aβ production [14].

The Rho GTPases Rac1, Cdc42, and RhoA play a critical role in macropinocytosis through actin polymerization and myosin light chain remodeling [16,28]. Rac1 is involved in the formation of the membrane ruffles and lamellipodia [29], while Cdc42 activation results in the generation of filipodia and the macropinocytic cup [28,30]. Furthermore, Rac1 can activate RhoA, which is important for the formation of stress fibers [31]. Arf6 has also been shown to bind to and activate both Rac1 and Cdc42 [32]. All together, these changes are believed to link plasma membrane receptor activation to the actin cytoskeleton to initiate, and carry out, macropinocytosis [28]. There is also evidence implicating these proteins in AD. Knockdown or dominant-negative mutations in Arf6 and Rac1 have been shown to decrease the amount of APP located in lysosomes [14]. Additionally, inhibiting either Rac1, RhoA, or Cdc42 has been demonstrated to reduce the production of Aβ [33,34].

However, the mechanism that links APP to these GTPases is unknown. An adaptor protein, Fe65, could link APP to these GTPases to internalize APP by macropinocytosis. The adaptor protein Fe65 is expressed in neurons and binds to the intracellular C-terminal/cytoplasmic YENPTY ‘endocytosis’ signal on APP [35], and binds to Arf6, resulting in both Arf6 and Rac1 activation [36,37]. Evidence from several studies has suggested that Fe65 functions as a scaffolding protein for actin regulators, by recruiting regulatory proteins to local sites requiring dynamic actin remodeling [37]. This study sought to examine whether Fe65, Arf6, Rac1, Cdc42 and RhoA are recruited to APP at the plasma membrane during its internalization by macropinocytosis. We also examined whether recruitment is sequential—Fe65 first, then Arf6, then Rac1, RhoA, and Cdc42, culminating in APP macropinocytosis.

Following APP binding/crosslinking with a fluorescent-labeled antibody, we observed the rapid recruitment of each protein to bound/crosslinked APP and membrane ruffles enriched with PI(4,5)P_2_. The recruitment of Rac1, Cdc42 and RhoA was sustained longer than Fe65 or Arf6, suggesting they may play prolonged roles downstream of these proteins. Further supporting their downstream role, the mutation of APP to prevent Fe65 binding or the pharmacological inhibition of Arf6 significantly reduced the recruitment of Rac1, Cdc42 and RhoA. Lastly, the antibody binding of APP was observed to rapidly increase the amount of GTP-bound Rac1, Cdc42, and RhoA, suggesting that increased recruitment coincides with increased GTPase activity. Together, these results demonstrate a network of regulatory proteins, which are recruited to the plasma membrane at sites of bound/crosslinked APP and are responsible for the trafficking of APP to lysosomes through macropinocytosis. Given that these small GTPases are readily targeted by small-molecule inhibitors, they could be therapeutic targets to reduce the production of Aβ peptides through the modulation of APP macropinocytosis.

## 2. Materials and Methods

### 2.1. Antibodies and Reagents

The antibodies purchased were mouse N-terminal anti-APP antibody (mAbP2-1, OMA1-03132, 1:100, Invitrogen, Waltham, MA, USA), mouse Aβ region anti-APP (6E10, 803001, 1:100, Biolegend, San Diego, CA, USA), rabbit anti-LAMP1 antibody (L1418, 1:200, Sigma-Aldrich, St. Louis, MO, USA), rabbit anti-NCAM1 antibody (EPR2187, ab220360, 1:100, Abcam, Waltham, MA, USA), rabbit anti-FE65 antibody (2877, Cell Signaling, Danvers, MA, USA), rabbit anti-Arf6 antibody (D12G6, 5740, Danvers, Cell Signaling, Danvers, MA, USA), Goat Anti-Rabbit-HRP conjugate (1706515, Bio-Rad, Mississauga, ON, Canada), and rabbit anti-GAPDH antibody (14C10, 2118, 1:1000, Cell Signaling, Danvers, MA, USA). The Zenon Alexa Fluor 647 Mouse IgG1 Labeling Kit (Z25008) and Zenon Alexa Fluor 647 Rabbit IgG Labelling Kit (Z25308) were purchased from Invitrogen (Waltham, MA, USA). The Zenon Alexa Fluor 647 Mouse IgG1 Labeling Kit (Z25008) and Zenon Alexa Fluor 647 Rabbit IgG Labelling Kit (Z25308) were purchased from Invitrogen (Waltham, MA, USA). The Arf6 inhibitor NAV-2729 (SML2238), Rac1 inhibitor EHT 1864 (E1657), Cdc42 inhibitor (SML0407), and Wortmannin (681675) were purchased from Sigma-Aldrich (St. Louis, MO, USA). The RhoA selective inhibitor Rhosin hydrochloride (5003) and ethylisopropyl amiloride (EIPA; 3378) were purchased from Tocris (5003; Bristol, UK). Pitstop2 was purchased from Abcam (ab120687; Waltham, MA, USA). Alexa488-conjugated transferrin was purchased from Invitrogen (T13342, Waltham, MA, USA). Further, 10 kDa and 70 kDa tetramethylrhodamine (TMR) conjugated dextran was purchased from Invitrogen (10 kDa—D1868, 70 kDa—D1818). Neuro-2a (N2a) mouse neuroblastoma cells were acquired from ATCC (CCL-131, Manassas, VA, USA). The reagents Dulbecco’s phosphate-buffered saline (DPBS, 14190144), Hank’s balanced salt solution (HBSS, 14025092) and trypsin-EDTA (25200056) were purchased from Gibco (CA, USA); fetal bovine serum (FBS, 090-150) from Wisent (Saint-Jean-Baptiste, QC, Canada); paraformaldehyde (PFA, 043368.9M) from Thermo Scientific (Waltham, MA, USA); and dimethyl sulfoxide (DMSO, D8418) from Sigma-Aldrich (St. Louis, MO, USA). G-LISA kits for GTP-bound Rac1 (BK128), Cdc42 (BK127) and RhoA (BK124) were all purchased from Cytoskeleton (Denver, CO, USA). Acti-stain 555 phalloidin was also purchased from Cytoskeleton.

### 2.2. DNA Constructs

The untagged APP695 (the full-length, neuronal isoform) construct was kindly provided by Dr. Jane Rylett (Robarts Research Institute, London, ON, Canada). APP695-AENATA (pCAX-APP-AENATA) was a gift from Dennis Selkoe and Tracy Young-Pearse (Addgene plasmid #30144). The generation of LAMP1 fused to mCherry (LAMP1-mCh) and CFP (LAMP1-CFP) constructs was described in a previous study by our lab [38]. Arf6-EGFP was a generous gift from Dr. Susan Meakin (Western University, London, ON, Canada). EGFP is fused to the C-terminal end of Arf6 due to the critical role of the N-terminus in membrane association [39]. Rac1-EGFP (pcDNA3.1-EGFP-Rac1(wt)) was a gift from Klaus Hahn (Addgene plasmid #13719) [40]. Cdc42-EGFP (pcDNA3.1-EGFP-Cdc42-wt) and RhoA-EGFP (pcDNA3.1-EGFP-RhoA-wt) was a gift from Gary Bokoch (Addgene plasmid #12975 and #12965) [41]. These Rho GTPase constructs are all N-terminal EGFP fusions and have been shown to have no effect on localization or function [40,41]. PLCδPH-mRFP was a generous gift from Dr. Bryan Heit (Western University, London, ON, Canada). Fe65-EGFP was a construct purchased from VectorBuilder (Chicago, IL, USA).

### 2.3. Cell Culture and Transfection

Neuro2a (N2a) cells were cultured in MEM containing 10% FBS in a 25 cm^2^ flask (130189, Thermo Scientific, Waltham, MA, USA) at 5% CO_2_ at 37 °C. The cells were passaged by trypsinization every 3–4 days. Once plated for experiments, cells were transiently transfected using Lipofectamine 2000 (11668019, Invitrogen, Waltham, MA, USA) according to the manufacturer’s protocol. After 24 h post-transfection, the N2a cells were differentiated by serum withdrawal (i.e., serum-free MEM) for 18 h. This differentiation method was chosen as serum withdrawal has been demonstrated to induce neurite outgrowth and the increased expression of neuronal protein markers such as NeuN [42,43]. Following differentiation, the cells were used for experiments as described below.

### 2.4. Co-Transfection of siRNA and Plasmids in N2a Cells

N2a cells were plated at a density of 1 × 10^5^ cells/well in 6-well plates or in 35 mm glass-bottom dishes, and cultured for 24 h prior to transfections. Cells were initially transfected with 50 nM on-TARGETplus siRNA targeting mouse ARF6 (L-043217-0005), FE65 (L-042929-01-0005), or with a scrambled non-targeting control siRNA (Dharmacon, Lafayette, CO, USA) using Lipofectamine 3000 (L3000015, Invitrogen, Waltham, MA, USA). After 24 h, these cells were subsequently transfected with LAMP1-mCherry and APP constructs using Lipofectamine 3000. Post transfection, the N2a cells were differentiated by serum withdrawal for 18 h. Differentiated cells were then used for APP internalization studies. The knockdown efficiency of ARF6 and FE65 was validated at both the transcript and protein levels by quantitative PCR (qPCR) and Western blot analysis, respectively.

### 2.5. Total RNA Isolation and qPCR

Total RNA was isolated from siRNA transfected N2a cells using TRIzol reagent (15596026, Invitrogen, Waltham, MA, USA). Briefly, cells were lysed in 500 μL of TRIzol and mixed with 100 μL of chloroform. Following a 10 min incubation at room temperature, samples were centrifuged at 12,000× *g* for 15 min at 4 °C. The aqueous phase was transferred to a fresh tube and mixed with an equal volume of isopropanol. RNA was precipitated by incubation at −20 °C for 30 min and followed by centrifugation at 12,000× *g* for 10 min at 4 °C. The RNA pellet was washed twice with 75% ethanol by centrifugation at 8000× *g* at 4 °C. The pellet was air-dried and resuspended in RNase free water.

For cDNA synthesis, 500 ng of total RNA was reverse-transcribed using iScript^TM^ cDNA synthesis kit (1708891, Bio-Rad, Mississauga, ON, Canada) according to the manufacture’s protocol. Quantitative real-time PCR (qPCR) was performed using 2 μL of cDNA, 0.5 μm of each of the forward and reverse primers specific for mouse ARF6 and FE65 (Table 1), and 5 μL of Sso AdvancedUniversal SYBR green Supermix (1725270, Bio-Rad, Mississauga, ON, Canada) in a final reaction volume of 10 μL. Amplification was carried out on a Bio-Rad CFX RT-PCR detection system using the following cycling conditions: initial denaturation at 95 °C for 10 s, followed by 35 cycles of 95 °C for 10 s and 60 °C for 15 s. The mouse RPL13α gene was used as the reference housekeeping gene (Table 1). Relative gene expression levels were calculated using the 2^−ΔΔCt^ method. The treatment of N2a cells with FE65 siRNA and ARF6 siRNA resulted in significantly lower fold changes in the expression level compared to scrambled control siRNA (Figure S1A,B), respective to their target.

### 2.6. Protein Extraction and Western Blotting

Total protein was extracted from N2a cells after 48 h following siRNA transfection using an ice-cold RIPA buffer containing the following ingredients: 50 mM Tris-HCl (pH 7.4), 150 mM NaCl, 1% NP-40, 0.5% sodium deoxycholate, and 0.1% SDS, along with a protease inhibitor cocktail. Briefly, cells were washed with PBS and lysed in 200 μL ice-cold RIPA buffer. Cell lysates were incubated on ice for 15 min with intermittent mixing and subsequent centrifugation at 14,000× *g* for 15 min at 4 °C. The resulting supernatant-containing soluble proteins were collected, and their concentration was determined using a BCA protein assay according to the manufacturer’s instructions. For protein analysis, 20 μg of total protein was separated using SDS-PAGE and transferred onto a PVDF membrane. The membrane was blocked in 5% non-fat dry milk and incubated overnight at 4 °C with primary antibodies against mouse Arf6 and Fe65. Following washing, membranes were incubated with appropriate HRP-conjugated secondary antibodies. The bands were visualized using chemiluminescence and imaged using a Chemidoc^TM^ MP imaging system (Bio-Rad, Mississauga, ON, Canada). Both FE65 siRNA and ARF6 siRNA resulted in the visible loss of Fe65 and Arf6 bands (Figure S1C,D), respectively.

### 2.7. Inhibitor Treatments

Following differentiation, cells were treated with inhibitors in fresh serum-free media. The small-molecule GTPase inhibitors EIPA and Pitstop2 were reconstituted in DMSO. As a vehicle control, 0.1% DMSO (*v*/*v*) in serum-free medium was used. For experiments examining the recruitment of the regulatory proteins of interest to crosslinked APP, the following concentrations and incubation times were used: 5 μM NAV-2729 (3 h), 10 μM EHT 1864 (18 h), 10 μM ML 141, 35 μM Rhosin (3 h), and 0.1% DMSO (18 h). Treatment with EHT and DMSO occurred overnight during the differentiation of N2a cells, and the remaining treatments were performed following differentiation. EHT 1864 and ML 141 concentrations were used in our previous study [38]. NAV-2729 and Rhosin treatments were chosen based on the previous literature [44,45], and a dose titration was performed for each to determine the doses used in this current study to inhibit the rapid internalization of APP to lysosomes (Figure S2). The Arf family of GTPases are also widely implicated in other cellular mechanisms, such as endocytic recycling [46]. Thus, we also assessed if Arf6 inhibition by NAV-2729 produced more broad changes in endocytosis at the concentration and treatment duration used by examining transferrin uptake. The NAV-2729 inhibition of Arf6 was not observed to significantly reduce the uptake of transferrin at the concentration and treatment duration used in this study (Figure S3).

Amiloride and amiloride-based compounds like EIPA are inhibitors of Na^+^/H^+^ exchangers (NHEs), and are commonly used to inhibit macropinocytosis. They have been shown to inhibit macropinocytosis by the downstream reduction of the activity of Rac1 and Cdc42 through local plasma membrane pH changes produced by blocking the activity of NHEs [47]. To assess its effects on APP internalization to lysosomes, cells were treated with 10 μM EIPA or 0.1% DMSO for 1 h in serum-free media. Pitstop2 was used to inhibit CME, as it has been shown to inhibit clathrin-coated pit formation [48]. This was chosen because dynamin inhibition using Dynasore has off-target effects and been shown to result in the inhibition of macropinocytosis [49]. Pitstop2 treatment was performed directly before crosslinking APP by incubating N2a cells with 20 μM Pitstop2 for 5 min. This treatment dose and timing was recommended by the manufacturer and validated by demonstrating reduced Alexa488 conjugated transferrin uptake in N2a cells following Pitstop2 treatment, as described (Figure S3).

### 2.8. Antibody-Mediated Cell-Surface APP Binding/Crosslinking

As performed in our previous studies on APP macropinocytosis [13,14,38], and by several other groups [24,50,51,52], the binding and/or crosslinking of APP was used to stimulate its internalization by macropinocytosis. For cell surface APP binding/crosslinking experiments, N2a cells were seeded at a density of 1.20 × 10^5^ cells in a 35 mm dish with a 14 mm glass bottom (P35G-1.5-14-C, MatTek, Ashland, MA, USA). Anti-APP (Invitrogen, Waltham, MA, USA) was labeled with Alexa Fluor 647 using a Zenon Alexa 647 Mouse IgG1 Labeling Kit (Invitrogen, Waltham, MA, USA) according to the manufacturer’s instructions. As a negative control, the anti-NCAM antibody (Abcam, Waltham, MA, USA) was labeled with Alexa Fluor 647 using a Zenon Alexa 647 rabbit IgG Labeling Kit (Invitrogen, Waltham, MA, USA) according to the manufacturer’s instructions and used to label cell surface NCAM. The labeled antibodies were incubated with N2a cells in HBSS on ice for 20 min. Previously, our lab has demonstrated that incubation with anti-APP antibodies on ice allowed for the thorough labeling of cell surface APP, but halted the cell’s ability to endocytose, and subsequent removal from ice stimulated the macropinocytosis of antibody-bound/crosslinked APP [14]. For a baseline measurement of regulatory protein recruitment, following incubation with antibodies on ice, cells were washed with ice-cold HBSS to remove unbound antibody and fixed on ice for 45 min with ice-cold 4% PFA (denoted as 0 min). For the 30 s time point, the cells were taken off the ice, washed once with HBSS pre-warmed to 37 °C to remove any unbound antibody, and immediately fixed (denoted as 30 s). For all other time points, after washing, warm HBSS was added to the cells, and they were incubated at 37 °C and 5% CO_2_ at indicated time points before fixing. Fixation for all time points (except 0 min) was performed by adding 4% PFA to cells for 15 min at room temperature. Cells selected for imaging had normal morphology and showed good expression of transfected constructs. All experiments were replicated three times, with at least 10 or 15 cells sampled for each condition at each time point.

### 2.9. Confocal Microscopy

Imaging was performed using a Leica SP8 confocal microscope with an HC PL APO CS2 63X 1.4 numerical aperture oil immersion lens (n_oil_ = 1.518). Individual cells were chosen based on morphology and protein expression, and zoom was applied to capture images of individuals cells. The resolution of the image was system-optimized to the zoom applied, and on average was optimized to approximately 512 × 512. The pinhole was set to 1AU, with images having an approximate average lateral and axial resolution of 0.4 μm 0.8 μm, respectively. Z-stacks were taken throughout the entire cell so bias would not be introduced in the selection of an individual plane during colocalization analysis. Z-stacks were set to a thickness of 0.3 μm. GFP fluorescence was imaged using a 488 nm excitation laser with a 500–550 nm filter set on an HyD hybrid detector. mRFP and mCherry fluorescence were visualized using a 552 nm excitation laser with a 570–620 nm filter on a PMT detector. Alexa Fluor 647 fluorescence was imaged using a 638 nm excitation laser with a 650–700 nm filter set on an HyD hybrid detector.

### 2.10. Data Quantification and Analysis

Colocalization analysis was performed on the z-stack images using Imaris 10.1.0 imaging software (Bitplane, South Windsor, CT, USA). In Imaris, colocalization is calculated on each individual z-plane in a z-stack image. To assess colocalization, we adapted and utilized a previously published approach to setting the colocalization threshold of the protein of interest to select only the brightest 0.5% of pixels in the channel of interest (adapted from [13]) to consider signal in an unbiased manner based on the intrinsic property of the image [13]. This specific value was chosen as these regulatory proteins have been previously shown to accumulate in sub-cellular locations where they are active [53]. Setting a strict threshold of the brightest 0.5% of pixels for each regulatory protein of interest ensured that changes in the colocalization of those brightest pixels could be captured. The threshold adopted for the other channel of interest (in most experiments this is LAMP1, crosslinked APP, or PLCδPH) was selected based on the average pixel intensity that demarcated a clear structure (vesicle or membrane ruffle) to ensure only clear signal was considered in the analysis. For example, to determine the recruitment of Arf6, the colocalization threshold for Arf6 was set to the brightest 0.5% pixels. This allowed for the quantification of changes in the accumulation of localized Arf6. To assess its colocalization with crosslinked APP, a threshold was set based on the average membrane signal intensity demarked by crosslinked APP. Imaris then generated the percentage colocalized by determining the number of pixels (above the threshold) of the two specified channels. Thresholded Mander’s correlation coefficient (MCC) was also provided for recruitment experiments to support the percentage colocalized. Using this unbiased method of measuring percentage colocalized, we compared the percentage colocalized between timepoints and conditions, all analyzed using the method described above. Graphing and statistical analysis were performed using Prism GraphPad 6.0 using either unpaired two-tailed *t*-tests or one-way ANOVAs with Tukey’s test. *p*-values of less than 0.05 were considered statistically significant.

### 2.11. Assessing Rho GTPase Activity Following APP Binding/Crosslinking Using G-LISA Assays

To assess for changes in active levels of Rac1, Cdc42, and RhoA in response to the antibody-mediated binding/crosslinking of APP, G-LISA assays were used. In short, a G-LISA is a commercially available 96-well plate-based immunosorbent assay that quantifies the amount of active GTP-bound GTPase by capturing only its GTP-bound form via an effector-coated plate, followed by antibody-based detection. For culturing cells for G-LISA assays, N2a cells were cultured in MEM containing 10% FBS in a 25 cm^2^ flask (130189, Thermo Scientific, Waltham, MA, USA) at 5% CO_2_ at 37 °C. The cells were passaged by trypsinization every 3–4 days. One day before transfection, cells were seeded at a density of 5.0 × 10^4^ cells in each well of a 4-well plate. Cells were subsequently transfected with the APP695 construct using lipofectamine 2000. The following day, the cells were serum-starved overnight for 18 h to ensure low basal levels of GTPase activity and induce differentiation. Following serum starvation, cells were placed on ice, and a 4-well plate was treated with anti-NCAM or anti-APP antibodies or with HBSS (no-treatment control). As a positive control, a 4-well plate was treated with EGF. Each 4-well plate was incubated with its respective treatment for 20 min on ice then moved to an incubator at 5% CO_2_, 37 °C for 2 min. Following this incubation, plates were immediately moved back to ice, washed two times with ice-cold PBS, and then the total protein was extracted according to the manufacturer’s protocol utilizing reagents included in the respective G-LISA kit. Protein concentration was measured by BCA to ensure equivalent amounts of total protein were loaded into each well of the G-LISA assay. Each G-LISA assay was run according to manufacturer protocol in technical and biological triplicates.

## 3. Results

### 3.1. N-Terminal APP Antibody-Mediated Binding/Crosslinking of Cell Surface APP Drives Internalization to Lysosomes Through Macropinocytosis

Our previous studies examining APP macropinocytosis utilized anti-HA or 6E10 anti-APP antibodies to bind/crosslink HA-tagged APP or untagged APP695 (the full-length human neuronal isoform) [13,14,38]. Here, we used an anti-APP antibody that targets an epitope spanning amino acids 104–118 of human APP within the GFLD [54] to bind a more physiologically relevant epitope. To ensure that binding/crosslinking with this antibody resulted in APP macropinocytosis, as we previously observed, we first compared the use of the N-terminal APP antibody with 6E10 [13,14,38]. N2a cells transduced with APP695 and LAMP1-mCherry (LAMP1-mCh) were incubated with fluorescent-labeled N-terminal APP antibody, 6E10 antibody, or NCAM antibody (negative control) on ice for 20 min, and fixed immediately (Time 0) or after incubation at 37 °C for 15 min. At Time 0, each antibody was observed entirely at the plasma membrane (Figure 1A–C). After 15 min, both the N-terminal APP (Figure 1A) and 6E10 (Figure 1B) antibodies demonstrated significantly higher colocalization between the tagged antibody and LAMP1 compared to NCAM (Figure 1C) after 15 min (Figure 1D; *p* < 0.001), demonstrating that N-terminal APP antibody binding/crosslinking rapidly directs APP to lysosomes, as observed with the 6E10 antibody.

To examine if the overexpression of LAMP1-mCh influenced the internalization of APP, we compared the colocalization of bound/crosslinked APP with LAMP1-mCh with that of endogenous immunolabeled LAMP1. There were no differences observed in the colocalization of APP between overexpressed LAMP1 or endogenous LAMP1 (Figure S4). We confirmed that these were lysosomes by labeling them via the overnight loading of LAMP1-labeled compartments with TMR-tagged 10 kDa dextran (Dex10), a technique commonly used to define terminal lysosomes [55]. Dex10 and LAMP1 signals appeared to significantly overlap, and no differences in APP colocalization were observed (Figure S5A,B). Additionally, APP co-internalized with 70 kDa dextran (Dex70; Figure S5C; a selective macropinocytosis marker) and was co-localized with LAMP1 [56].

To confirm that this rapid uptake was due to macropinocytosis, the uptake of bound/crosslinked APP was examined following treatment with commonly used inhibitors of macropinocytosis, EIPA (NHE inhibitor) or wortmannin (PI3K inhibitor), and compared to vehicle control (DMSO) or treatment with Pitstop2 (clathrin coat inhibitor). The inhibition of NHEs has been shown to prevent the local membrane pH changes required for macropinocytosis [47], and PI3K has been shown to regulate growth factor-mediated macropinocytosis [57]. Both treatments significantly reduced APP colocalization with LAMP1 compared to treatment with DMSO or the CME inhibitor Pitstop2 (Figure 1E,F; *p* < 0.001).

### 3.2. Fe65 and Arf6 Are Rapidly and Transiently Recruited to Bound/Crosslinked APP and Membrane Ruffles

Antibody-mediated APP binding/crosslinking was then utilized to assess the recruitment of Fe65. N2a cells were transduced with APP695, Fe65-EGFP and the ruffling membrane marker PLCδPH-mRFP. PLCδPH is frequently used to label membrane PI(4,5)P_2_, which is enriched transiently in membrane ruffles and contributes to initiating macropinocytosis [58]. Transduced cells were incubated with fluorescently labeled anti-APP or anti-NCAM antibodies on ice, and then either fixed immediately (‘Time 0′ or ‘baseline’) or after incubation at 37 °C for 30 s and 2, 5, and 10 min. Fe65 was rapidly recruited to bound/crosslinked APP within 30 s, as demonstrated by an increase in colocalization (Figure 2A). In comparison, no discernible changes in colocalization were observed in response to NCAM binding/crosslinking (Figure S6A). The rapid colocalization of Fe65 also occurred with PLCδPH after 30 s (Figure 2A). Significantly larger changes in percent colocalized and MCC were demonstrated at 30 s between Fe65 and bound/crosslinked APP (8.68 ± 0.484; *p* < 0.001; Figure 2B) or PLCδPH (9.16 ± 0.292; *p* < 0.001; Figure 2C) compared to baseline. When NCAM was bound/crosslinked, there were no significant changes in colocalization at any timepoint compared to baseline (Figure 2B,C). When comparing APP to NCAM binding/crosslinking at each time point, a significantly higher difference from baseline was observed at 30 s for both colocalization measurements with APP (*p* < 0.001; Figure 2B,C).

Next, to assess the recruitment of Arf6 upon APP binding/crosslinking, N2a cells were transduced with APP695, Arf6-EGFP and PLCδPH and incubated with fluorescently labeled APP or NCAM antibodies, as described above. Changes in the colocalization of Arf6 with APP or PLCδPH demonstrated a pattern similar to Fe65, with a peak in colocalization between Arf6 and bound/crosslinked APP or PLCδPH observed within 30 s (Figure 2D). No changes in the amount of colocalization between Arf6 and labeled antibody or PLCδPH were observed in response to NCAM binding/crosslinking (Figure S6B). A significantly larger difference from baseline was observed at 30 s with APP binding/crosslinking when analyzing colocalization between Arf6 and anti-APP (29.4 ± 1.54; *p* < 0.001; Figure 2E) and PLCδPH (18.7 ± 1.02; *p* < 0.001; Figure 2F). No significant changes in colocalization from baseline were observed within anti-NCAM-treated timepoints (Figure 2E,F). At 30 s, anti-APP treatment demonstrated a significantly larger difference from baseline than anti-NCAM treatment for Arf6 colocalization with tagged antibody (*p* < 0.001; Figure 2E) or PLCδPH (*p* < 0.001; Figure 2F).

### 3.3. Rac1, Cdc42 and RhoA Demonstrate Rapid and Sustained Recruitment to Bound/Crosslinked APP and Membrane Ruffles

Next, the recruitment of the Rac1, Cdc42 and RhoA during APP macropinocytosis was assessed. Cells expressing APP695, Rac1-EGFP and the ruffling membrane marker PLCδPH-mRFP were incubated with fluorescently labeled APP or NCAM antibodies on ice, and then either fixed immediately (Time 0) or after incubation at 37 °C for the time points outlined above. The increased colocalization of Rac1 was observed with bound/crosslinked APP and PLCδPH at 30 s (Figure 3A), as was observed with Fe65 and Arf6. However, recruitment was sustained, as increased colocalization continued to be observed at 2 min (Figure 3A). The binding/crosslinking of NCAM produced no visible changes in colocalization with either tagged antibody or PLCδPH (Figure S6C). Significantly higher differences in the percentage colocalized or MCC of Rac1 with anti-APP antibody were observed at 30 s (29.44 ± 3.720; *p* < 0.001) and 2 min (24.87 ± 3.720; *p* < 0.001) compared to baseline (Figure 3B). The same was observed with the colocalization between Rac1 and PLCδPH at 30 s (26.65 ± 7.072; *p* = 0.009) and 2 min (19.48 ± 7.072; *p* = 0.05; Figure 3C).

Similar observations were made when examining the recruitment of Cdc42, with colocalization visually peaking at 30 s and 2 min following APP binding/crosslinking (Figure 3D). As expected, there was no change in colocalization observed across any timepoint in response to NCAM binding/crosslinking (Figure S6D). A significantly higher difference from baseline was observed in the colocalization between Cdc42 and bound/crosslinked APP at 30 s (22.4 ± 1.86; *p* < 0.001) and 2 min (21.7 ± 1.89; *p* < 0.001; Figure 3E). However, the difference in the percentage of Cdc42 colocalized to baseline remained significantly increased between Cdc42 and PLCδPH at all timepoints (Figure 3F)—30 s (24.1 ± 2.72), 2 min (23.9 ± 2.29; *p* < 0.001), 5 min (14.1 ± 1.70; *p* < 0.001) and 10 min (11.6 ± 2.80; *p* < 0.001)—while the change in MCC values was only significantly higher at 30 s and 2 min (Figure 3F). No differences were observed between any timepoint when NCAM was bound/crosslinked, and the difference from baseline was significantly higher for anti-APP treatment compared to anti-NCAM at 30 s and 2 min for both colocalization measurements assessed (*p* < 0.001; Figure 3E,F).

For RhoA, we observed increased colocalization with bound/crosslinked APP or PLCδPH at 30 s and 2 min, which decreased at 5 and 10 min (Figure 3G). No changes in colocalization over time were observed in response to the binding/crosslinking of NCAM (Figure S6E). The quantification of the difference in colocalization between each time point and baseline revealed a significant increase in RhoA–anti-APP colocalization from baseline at 30 s (11.3 ± 1.54; *p* = 0.002) and 2 min (9.74 ± 0.342; *p* = 0.02; Figure 3H). For the colocalization of RhoA with PLCδPH, a significantly higher difference from baseline in the percentage colocalized was observed in response to APP binding/crosslinking at 30 s (10.4 ± 0.576; *p* < 0.001), 2 min (7.80 ± 0.494; *p* < 0.001) and 5 min (4.08 ± 3.46; *p* = 0.004; Figure 3I). However, the MCC between RhoA and PLCδPH only remained significantly higher than baseline at 30 s (Figure 3I). No significant increases or decreases were observed in any colocalization measure in NCAM antibody conditions (Figure 3H,I).

We also examined the activation of Rac1, Cdc42, and RhoA following antibody binding using a G-LISA kit (Cytoskeleton) to quantify active GTP-bound Rac1, Cdc42 and RhoA. To do so, cells expressing APP695 were incubated with anti-APP antibodies on ice and then incubated for 2 min to allow the stimulation of APP macropinocytosis. Lysates were immediately collected from cells and were used to assess the levels of GTP-bound Rac1, Cdc42 and RhoA by G-LISA. As a control, this experiment was also run in untreated cells and cells incubated with anti-NCAM antibodies (negative control) or EGF (positive control). A significantly higher absolute optical density was measured in response to both APP binding/crosslinking and positive control compared to either NCAM binding/crosslinking or untreated cells in Rac1 G-LISAs (*p* < 0.001; Figure 3J), Cdc42 G-LISAs (*p* < 0.001; Figure 3K), and RhoA GLISAs (*p* < 0.001; Figure 3L). Increased absolute optical density measurements in response to antibody-mediated APP binding/crosslinking indicate higher levels of GTP-bound or active Rac1, Cdc42 and RhoA.

Given the increased GTPase activity and increases in colocalization of these Rho GTPases with APP and PLCδPH through both 30 s and 2 min, we examined whether bound/crosslinked APP and PLCδPH localize with actin at the plasma membrane at these time points. We also sought to examine if PLCδPH-rich membrane domains contained high amounts of actin, as would be expected with membrane ruffles. At both 30 s (Figure S7A) and 2 min (Figure S7B), both APP and PLCδPH can be visualized at the plasma membrane overlapping with phalloidin-labeled F-actin.

### 3.4. Mutation of APP Intracellular Domain YENPTY Sequence and siRNA Knockdown of Fe65 Prevents the Rapid Macropinocytosis of APP

The C-terminal YENPTY sequence on APP has been shown to mediate the binding of Fe65 [59]. To examine the role of this binding site in the macropinocytosis of APP and the recruitment of GTPases, N2a cells were transduced with LAMP1-mCh and either wildtype APP695 (WT-APP) or a mutant APP, in which the YENPTY sequence is mutated to AENATA (APP-AENATA). APP was bound/crosslinked by N-terminal APP antibodies while on ice and then allowed to internalize over a 15 min incubation. While WT-APP internalized to LAMP1-labeled compartments, as expected, APP-AENATA remained at the membrane (Figure 4A) and colocalization with LAMP1 was significantly lower in AENATA-expressing cells (*p* < 0.001; Figure 4B). We also assessed the effect of the siRNA-mediated knockdown of FE65 expression on APP macropinocytosis. To do so, scramble control siRNA or FE65 siRNA-treated N2a cells were transduced with APP695 and LAMP1. After this, APP was bound/crosslinked and allowed to internalize for 15 min to assess the effects of the treatment on internalization to LAMP1-labeled lysosomes. In FE65 siRNA treated cells, APP is observed primarily at the membrane after 15 min (Figure 4C), similar to APP-AENATA-expressing cells. Colocalization with LAMP1 was significantly lower with FE65 siRNA treatment compared to the control siRNA treatment (*p* = 0.001; Figure 4D). Together, these results suggest that preventing Fe65 binding by mutating the YENPTY sequence of APP inhibits the macropinocytosis of APP.

### 3.5. Mutation of APP Intracellular Domain YENPTY Sequence Reduces Recruitment of Fe65, Arf6, and the Rho GTPases Rac1, Cdc42 and RhoA

Next, we examined the effects of YENPTY sequence mutation on regulatory protein recruitment to bound/crosslinked APP 30 s after antibody-mediated binding/crosslinking. First, investigating Fe65 recruitment, the binding/crosslinking of APP-AENATA resulted in significantly lower percentages of Fe65 colocalized with both crosslinked APP (3.52% ± 0.674; *p* < 0.001) and PLCδPH (6.84% ± 0.640; *p* < 0.001) compared to WT-APP (Figure 5A,B). Arf6 recruitment was also reduced with the mutation of the YENPTY sequence, with the cells transfected with APP-AENATA showing a significantly lower percent of Arf6 colocalized with bound/crosslinked APP (−13.6% ± 1.31; *p* < 0.001) and PLCδPH (−19.8% ± 1.35; *p* = 0.002) compared to WT-APP (Figure 5C,D). Similar findings were observed for Rac1, Cdc42 and RhoA. APP-AENATA-expressing cells showed lower Rac1-APP colocalization (−14.9 ± 2.60; *p* = 0.005) and Rac1-PLCδPH colocalization (−23.99 ± 3.137; *p* = 0.002) than WT-APP (Figure 5E,F). The percent colocalization of Cdc42 with APP (−22.31 ± 3.103; *p* = 0.002; Figure 5G,H) or PLCδPH (−28.45 ± 3.385; *p* = 0.005; Figure 5G,H), as well as RhoA with APP (−10.34 ± 2.187; *p* = 0.009; Figure 5I,J) or PLCδPH (−14.61 ± 2.883; *p* = 0.007; Figure 5I,J), was significantly lower as a result of APP-AENATA.

### 3.6. The Recruitment of Fe65 and Arf6 to Bound/Crosslinked APP and Membrane Ruffles Is Reduced in Response to Arf6 Inhibition

To further investigate the proposed signaling cascade, we explore whether the inhibition of the activity of individual small GTPases influences the recruitment and activation of other non-targeted GTPases. First, we assessed the effect of treatment with each inhibitor on APP macropinocytosis to lysosomes 15 min after APP binding/crosslinking. The treatment with the Arf6 inhibitor NAV-2729 resulted in APP being primarily observed at the membrane, with significantly less colocalization observed with LAMP1 (Figure 6A,B). To confirm that this result was not due to off-target effects from the inhibitor, we also examined the effect of Arf6 knockdown on APP macropinocytosis. Similar to NAV-2729 treatment, APP internalization was visibly reduced and colocalization with LAMP1 significantly decreased with Arf6 knockdown (Figure 6C,D). Reduced internalization and significantly decreased colocalization with LAMP1 was also observed with the RhoA-specific inhibitor Rhosin (Figure 6E,F). Additionally, we confirmed that treatment with the Rac1 inhibitor EHT 1864 and Cdc42 inhibitor ML 141 prevented APP macropinocytosis and reduced LAMP1 colocalization (Figure 6G,H), as we have previously reported [38].

Next, to investigate if the recruitment of each of these proteins is sequential, each GTPase was inhibited to examine the effects on the recruitment of each protein of interest at 30 s when colocalized with bound/crosslinked APP peaks, as described above. N2a cells were transduced as previously described and treated with inhibitors for Arf6 (NAV-2729), Rac1 (EHT 1864), Cdc42 (ML 141) or RhoA (Rhosin). APP was bound/crosslinked by the antibody on ice, incubated at 37 °C for 30 s, then fixed and imaged. Based on studies that have demonstrated that Fe65 acts as a scaffolding protein and recruits Arf6 and Rac1 for actin polymerization [36,37], we predicted that treatment with any GTPase inhibitors tested would have no effect on Fe65 recruitment. Surprisingly, Arf6 inhibition did significantly reduce the percent of Fe65 colocalized with APP (−8.060 ± 1.392; *p* = 0.001) and PLCδPH (−12.01 ± 1.987; *p* < 0.001) compared to the DMSO-treated vehicle control, with no significant reductions being observed with Rac1, Cdc42 or RhoA inhibition (Figure 7A,B).

With Arf6 previously shown to mediate the recruitment and activation of Rac1 in macropinocytosis [32], we expected that it acts upstream of the Rho GTPases, and predicted that only the inhibition of Arf6 itself would result in changes to its recruitment to bound/crosslinked APP and membrane ruffles. As predicted, Arf6 inhibition reduced both its colocalization with APP (−25.35 ± 3.525; *p* < 0.001) and PLCδPH (−20.88 ± 3.506; *p* < 0.001) compared to DMSO vehicle control (Figure 7C,D). However, Rac1 inhibition (−15.10 ± 3.252; *p* = 0.006) and RhoA inhibition (−16.67 ± 3.252; *p* = 0.003) also reduced the percent of Arf6 colocalized with bound/crosslinked APP (Figure 7D), albeit to a lesser extent than Arf6 inhibition. The significant reduction in Arf6 within PLCδPH-labeled membrane ruffles was also observed with Arf6 inhibition (−20.88 ± 3.506; *p* < 0.001; Figure 7C,D) and RhoA inhibition (−13.92 ± 3.506; *p* = 0.02; Figure 7C,D).

### 3.7. The Recruitment of the Rho GTPases Rac1, Cdc42 and RhoA to Bound/Crosslinked APP and Membrane Ruffles Demonstrate Differing Responses to GTPase Inhibition

Examining the effect of GTPase inhibition on the recruitment of Rac1, Cdc42 and RhoA, we found Rac1 colocalization with bound/crosslinked APP was significantly reduced following treatment only with the Arf6 inhibitor (−30.33 ± 5.503; *p* < 0.001; Figure 8A,B) compared to the vehicle control. Only Arf6 inhibition had a significant effect on the colocalization between Rac1 and PLCδPH (−27.71 ± 5.713; *p* = 0.002; Figure 8A,B). In Cdc42-expressing cells, Arf6 inhibition (−27.46 ± 3.679; *p* < 0.001), Rac1 inhibition (−17.68 ± 3.679; *p* = 0.005), and RhoA inhibition (−17.53 ± 3.679; *p* = 0.005) all demonstrated significant reductions in Cdc42-APP colocalization (Figure 8C,D). However, only Arf6 inhibition significantly reduced the colocalization of Cdc42 with PLCδPH (−23.32 ± 4.363; *p* = 0.002; Figure 8C,D). RhoA colocalization with either bound/crosslinked APP or PLCδPH was also significantly reduced compared to vehicle control in response to treatment with all the inhibitors examined (*p* < 0.01; Figure 8E,F).

To assess if any of the inhibitors used produced off-target effects on a non-targeted GTPase, we examined the effects of each inhibitor at the concentrations used above on basal Rac1, Cdc42 and RhoA activity using Rac1, Cdc42 and RhoA G-LISA assays in non-transduced N2a cells. Only treatment with EHT 1864 resulted in significantly reduced GTP-bound Rac1 (*p* < 0.05; Figure S8A). Likewise, only treatment with the inhibitor ML 141 resulted in a significant reduction in the amount of GTP-bound Cdc42 (*p* < 0.05; Figure S8B). Levels of RhoA-GTP were observed to only be significantly reduced in response to treatment with the inhibitor Rhosin (*p* < 0.05; Figure S8C).

## 4. Discussion

The internalization of APP is a key step in the production of Aβ. Utilizing an antibody to bind/crosslink APP and stimulate APP macropinocytosis, we have demonstrated the recruitment of Fe65, Arf6, Rac1, Cdc42 and RhoA to bound/crosslinked APP and PI(4,5)P_2_-enriched membrane ruffles labeled by PLCδPH. Fe65 and Arf6 were recruited rapidly and transiently, demonstrated by peak colocalization at 30 s and return to baseline at 2 min. Rac1, Cdc42 and RhoA were also rapidly recruited at 30 s; however, recruitment was sustained over 2 min. Together, these results may suggest an earlier role for Fe65 and Arf6 recruitment in the activation of APP macropinocytosis, with the recruitment of the Rho GTPases downstream of Fe65 and Arf6 and involved more directly in the actin remodeling required to carry out macropinocytosis. This is further supported by the observation that the mutation of YENPTY sequence and Arf6 inhibition prevented the recruitment of all regulatory proteins investigated, supporting that Fe65 and Arf6 may have an upstream effect on Rac1, Cdc42 and RhoA. This would align with previous studies, which demonstrated that Fe65 binds directly to Arf6 resulting in Arf6 and Rac1 activation [36,37], and Arf6 can bind to and activate Rac1 and Cdc42 via Arf6-ARNO interaction [32].

It must be noted that there were observed effects of GTPase inhibitors on recruitment that were not expected given our proposed cascade. This includes our observation that Arf6 inhibition resulted in decreased Fe65 recruitment, and Rac1 inhibition resulted in decreased Arf6 recruitment. This could suggest that the regulatory relationship between these proteins is bidirectional. However, it could be possible that the recruitment of Fe65 and Arf6 could occur prior to 30 s but quickly dissociate without the downstream changes produced by Rho GTPase activity. This is a possible explanation, given that macropinocytosis is known to be tightly regulated by the coordination of changes in PI phosphorylation states with the activity of many regulatory proteins, including the Rho GTPases [21]. This coordination is exemplified by the direct interactions observed between Arf6 and phosphatidylinositol 4-phosphate 5-kinase alpha (PI(4)P5Kα) [60], or Rac1 and PI3Kβ [57]. To explain some of the unexpected results of this study and fully characterize the regulation of APP macropinocytosis, future studies should examine the coordination of PIPs with GTPases in live cells, investigate the role of GEF/GAP proteins, and demonstrate the direct molecular interaction between regulatory elements, including the proteins examined in this study.

There are limitations to this study that are important to highlight, and we should discuss their implications regarding the interpretation of the results. One of these is the use of only the N2a cell line in the experiments described above. This cell line was chosen for its technical advantages, given its ease of use and the ability to generate a large number of cells that enable the acquisition of many images from several timepoints across replicates. N2a cells were also selected over other neuronal transformed cell lines, since they do not contain any mutations in genes that are known to be involved in macropinocytosis. However, the use of this cell line comes with limitations given that it originates from mice and is not a primary cell. The limitation of using a cell line from mice that expresses mouse APP was mitigated by the transfection of human APP695 (the isoform primarily expressed in neurons) and visualizing/stimulating the macropinocytosis of APP695 by binding/crosslinking using an antibody targeting residues within the growth factor-like domain (GFLD) of human APP. Although these steps were taken, the fundamental physiological differences between transformed neuronal cell lines and primary neurons are still a limitation to consider. While studies have not yet been conducted on human primary neurons, we have previously utilized the antibody-mediated binding/crosslinking of human APP to investigate APP trafficking in mouse primary neurons. In this previous work, we demonstrated the macropinocytosis of APP to lysosomes in mouse primary neurons, which was reduced by the expression of a dominant-negative Arf6 mutant [14], or by the Rac1 and Cdc42 inhibitors used in the current study [38]. While these results support the observations made in the current study, the macropinocytosis of APP needs to be investigated further in human primary neurons.

Altogether, our results provide the first evidence, to our knowledge, of a network of proteins that is recruited and activated to regulate the macropinocytosis of APP. Along with our previous observations [13,14,38], these results continue to suggest a possible growth-factor like function of APP, involving the stimulation of macropinocytosis following its binding and/or crosslinking, and can result in the production of Aβ in lysosomes. The results of the current study also provide additional support for our prior observations that dominant negative Arf6 mutations, the small-molecule inhibition of Rac1, RhoA and Cdc42, and the siRNA knockdown of all of these GTPases result in reduced APP macropinocytosis and Aβ production [14,38]. In our previous studies, we had only demonstrated that the activity of Arf6, Rac1, Cdc42 and RhoA was required for the rapid internalization of APP to lysosomes.

Building upon this work, in the current study, we aimed to further elucidate the role the activity of these proteins plays in regulating the macropinocytosis of APP, and its subsequent cleavage to Aβ once internalized to lysosomes. Here, we have provided evidence that these proteins mediate this by their recruitment to APP, demonstrated by the observed colocalization with bound/crosslinked APP and PI(4,5)P_2_ enriched membrane ruffles. While the results demonstrate that the recruitment and activity of the proteins investigated are both involved in the regulation of APP macropinocytosis, it needs to be made clear that these experiments are limited in their ability to examine direct binding between proteins. Previous studies have established direct binding between some members of the proposed cascade in other processes. As described above, Fe65 binds to APP via the YENPTY sequence within its extracellular domain [59]. It has also been shown to bind to and activate Arf6 to regulate actin polymerization [36,37]. This taken together might suggest that direct interactions between members of the proposed cascade occur; however, future studies need to confirm this via experiments that directly measure molecular interactions, such as co-immunoprecipitation or proximity ligation assays.

These results also provide further information about the underlying mechanisms of previous studies linking Arf6, Rac1, Cdc42 and RhoA to the production of Aβ. For example, a previous study demonstrated that the inhibition of Rac1 and ROCKII, a downstream effector of RhoA, decreased Aβ40 and Aβ42 production in vivo [33,34]. It now seems likely that the underlying mechanism behind these past findings involves the inhibition of APP macropinocytosis, considering the results of the current study and our previous work [14,38]. Additionally, our collective findings on APP macropinocytosis suggest that it could be the underlying mechanism responsible for the findings of altered levels and distributions of RhoA in AD patients [61], the increased hippocampal activity of Rac1 in AD brains [62], and the upregulation of Arf6 expression in the hippocampus of AD patients [14].

To develop a concept of the possible physiological role of APP macropinocytosis, comparing the separate bodies of literature regarding the cellular physiology of APP and macropinocytosis yields interesting connections. Both APP and macropinocytosis have been linked to growth cone function, axonal extension and turning, and are broadly connected to synaptic plasticity. APP has been shown to play a role in the formation of synapses. Knock-outs of APP have demonstrated that it plays an important role in long-term potentiation (LTP) and synaptic plasticity in the adult mouse brain [63]. On the cellular level, it has been observed that APP is enriched in both growth cones and synapses [25,26], where it regulates neurite and growth cone formation through interactions with various stimulating factors such as neural growth factor (NGF) [25] and Netrin-1 [64]. Our observations supporting the regulatory function of Fe65 and Arf6 in APP macropinocytosis may provide insights linking these previous studies. Arf6 has been shown to mediate neurite outgrowth through Fe65 [36], and Fe65 itself is enriched in growth cones and synapses [26]. Furthermore, the regulatory roles of Rac1, Cdc42 and RhoA in neurite outgrowth and growth cone formation have also been demonstrated [65]. However, a direct investigation into how APP mediates its role in growth cone activity and synapse formation, and specifically whether APP macropinocytosis is involved, has not yet been conducted.

Although APP was sequenced in 1987 [18], its role remains unknown. APP was suggested to act as a cell surface receptor since its identification, and studies of its structure have revealed domains that can mediate ligand binding and the formation of APP dimers [54]. The results demonstrated in this study, along with our previous findings, suggest that APP acts as a cell surface receptor, stimulating its macropinocytosis, and that it could be linked to its role in physiology, as described above. This is analogous to growth factor receptors, where ligand binding can drive the homodimerization of growth factor receptors in response to ligand binding and subsequent C-terminal phosphorylation, resulting in growth-factor-induced macropinocytosis [21,57]. Given the presence of the GFLD in the E1 domain of APP [54], a similar receptor-mediated mechanism could be involved in APP macropinocytosis, and this would explain the stimulation of APP macropinocytosis by antibody-mediated binding/crosslinking, as used in this study. This domain demonstrates a high degree of structural similarity with growth factor receptors, and the broader E1 domain has been demonstrated to bind to a number of extracellular proteins and potential APP ligands [54,66]. Potential ligands include Reelin and NGF [19], which have been shown to stimulate macropinocytosis within growth cones [67]. Further, in other cell surface receptors, the YENPTY sequence and other similar sequences have been shown to regulate the recruitment proteins that regulate macropinocytosis in response to ligand binding [21,68]. This model of APP-stimulated macropinocytosis could further explain the previously discussed links between APP and macropinocytosis in growth cones.

Consideration of what is known about the structure and function of APP also yields interesting links between AD pathological mechanisms and the methods used here to stimulate APP macropinocytosis by its binding/crosslinking. In addition to proposed receptor functions, APP has also been suggested to act as a trans-synaptic adhesion protein through the formation of trans-dimers across the synapse [69,70]. However, when not forming trans-dimers across the synapse, APP can be found in the membrane as cis-dimers or homodimers [71]. Previous work has demonstrated that the formation of APP homodimers has been shown to increase the production of Aβ [20,22,23]. Additionally, the dimerization of APP has been shown to increase the phosphorylation of the intracellular YENPTY sequence, specifically at the first tyrosine residue (Y682) [72]. This is the sequence that the second phosphotyrosine binding domain (PTB2) of Fe65 binds to in the presence of plasma membrane PI(4,5)P_2_ [59]. The phosphorylation at Y682 has resulted in the increased trafficking of APP to acidic compartments and the production of Aβ [73]. If these results are considered with the findings of the current study using antibody-mediated APP binding/crosslinking, the dimerization of APP may serve as a mechanism that drives Aβ production through the macropinocytosis of APP to lysosomes. Supporting this, the increased phosphorylation of APP at Y682 has been observed in neurons derived from AD patients [74], and the immunostaining of Fe65 in AD patients has been shown to increase with disease severity [75]. However, which pathological changes in AD result in increased APP dimerization or macropinocytosis remains unknown.

Given the design and methodology of this study, several questions do remain regarding how APP macropinocytosis is stimulated and regulated. With the labeling of cell surface APP by FlAsH-EDT_2_ being previously demonstrated to traffic to Rab-5-labeled early endosomes [14], it appears that rapid APP macropinocytosis to lysosomes may be specific to the ligand binding/crosslinking of APP. Supporting our findings using this approach, the antibody-mediated binding/crosslinking of APP has been utilized by several other labs, which also demonstrated the increased endocytosis of APP as a result [24]. The mechanism by which the use of antibodies may stimulate this remains unclear. Our results suggest that this may not be due to the specific epitope targeted, as both extracellular Aβ region- and GFLD domain-targeting antibodies resulted in APP macropinocytosis. It might be possible that bivalent binding by these antibodies could bring two APP molecules together, resulting in the formation of an APP homodimer. Structurally, both APP antibodies we have used for stimulating APP macropinocytosis bind to regions containing or adjacent to domains that can mediate APP dimerization [19]. The underlying mechanism remains unclear, and our results are limited to the broad description of it as binding and/or crosslinking. Examining the underlying mechanism more precisely could prove to better characterize the role of APP in its macropinocytosis, and aid in identifying an endogenous ligand that stimulates APP macropinocytosis.

Our results utilizing the YENPTY sequence mutation of APP also raise several questions. First, it should be noted that the dimerization of APP, and the phosphorylation of tyrosine residues within the YENPTY sequence, have been implicated in other endocytic mechanisms, including clathrin-mediated endocytosis (CME). The phosphorylation of the first tyrosine of the YENPTY sequence was required for the immunoprecipitation of APP with adaptor protein-2 (AP2) and clathrin [76]. It is worth noting that internalization by both CME and macropinocytosis has been observed in many growth factor receptors [77]. This seems highly likely given several publications have provided evidence for the internalization of APP by CME [76,78,79]. The examination of the role of YENPTY sequence in determining the endocytic mechanism that internalizes cell surface APP across various contexts is needed to better reconcile these differing findings. This includes its specific function in the regulation of APP macropinocytosis. Our findings could be taken to suggest that its role is to mediate the binding of Fe65 to APP, given its mutation prevented the recruitment of Fe65 to bound/crosslinked APP. However, several other proteins bind to this sequence, including JIP1b, X11/Mint and Dab1 [80]. Without a clear demonstration that the depletion of Fe65 prevents APP macropinocytosis, its regulatory role in the process remains unclear.

It is becoming clear that gaining a greater understanding of the trafficking of APP from the plasma membrane is going to be critical to understanding the physiological function of APP and its role in AD. A considerable amount of evidence has clearly demonstrated that the production Aβ is intimately tied to its endocytosis [81,82]. Across multiple studies, we have shown that APP is internalized by macropinocytosis to lysosomes and increases Aβ production. We have also examined some of the regulatory mechanisms involved and linked these back to the production of Aβ [14,38]. However, it is also clear that other mechanisms are involved in the endocytosis of APP. The characterization of the contexts that drive APP internalization through different endocytic mechanisms is desperately needed, and will require the comparison of these mechanisms in both normal physiology and AD. Doing so would provide critical insights into the physiological function of APP and what causes the overproduction of Aβ in AD.

## 5. Conclusions

Here, we have demonstrated the recruitment of the known macropinocytosis regulatory proteins Arf6, Rac1, Cdc42, and RhoA to bound and/or crosslinked APP. Further, we have provided evidence of a potential role of Fe65 in acting as a scaffolding protein for the recruitment of these proteins to bound/crosslinked APP. Together, these observations demonstrate a network of regulatory proteins recruited to APP that controls the internalization of APP to lysosomes by macropinocytosis. Since GTPases are readily targeted by pharmacological inhibitors, this network could be explored as a novel avenue for therapies aimed at reducing Aβ production by reducing the trafficking of APP from the cell surface to lysosomes by macropinocytosis.

## Figures and Tables

**Figure 1:**
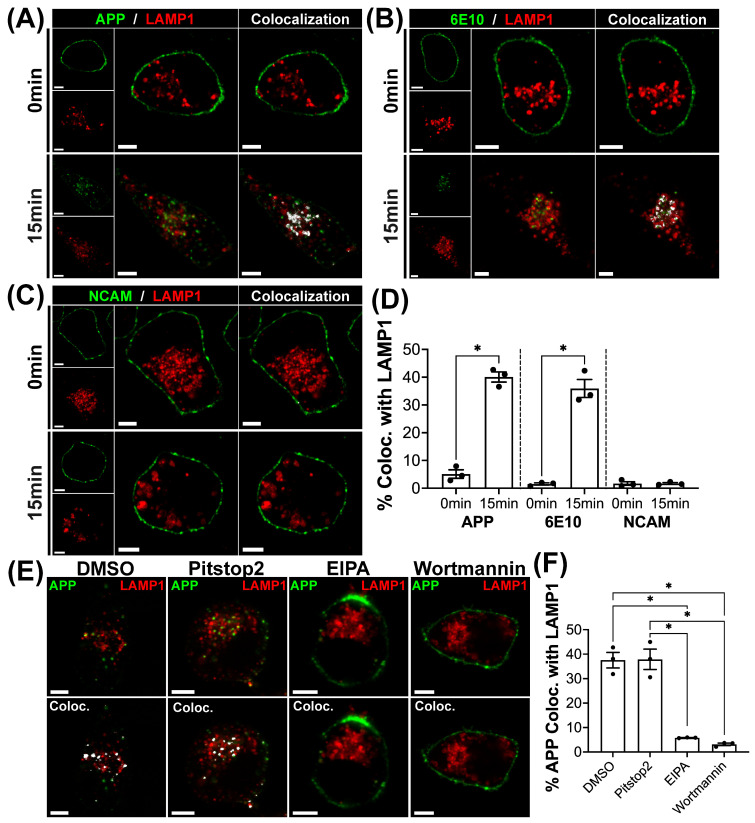
The N-terminal anti-APP antibody-mediated binding/crosslinking of APP results in rapid internalization to LAMP1-labeled compartments by macropinocytosis. N2a cells transfected with APP695 and LAMP1-mCh (red) and incubated with fluorescent-tagged (**A**) N-terminal anti-APP (APP), (**B**) Aβ-region targeting 6E10 antibodies, or (**C**) anti-NCAM antibodies (green) on ice for 20 min, then immediately fixed or incubated at 37 °C for 15 min, fixed and imaged by confocal microscopy. Colocalization was quantified between antibody-bound APP and LAMP1 using Imaris 10 to generate a colocalization channel (white pixels). (**D**) Quantification of the mean % of APP colocalized with LAMP1 from three replicate experiments (*n* = 3 independent experiments; 10 images acquired for each condition per replicate), with significance calculated by a one-way ANOVA with Tukey’s test. (**E**) N2a cells transfected with APP695 and LAMP1-mCh (red) that were treated with DMSO, 20 μM Pitstop2, 10 μM EIPA, or 300 nM wortmannin. Cells were imaged 15 min after APP binding/crosslinking. Colocalization was assessed between crosslinked APP and LAMP1 (white pixels). (**F**) Quantification of the mean % of APP colocalized with LAMP1 (*n* = 3 independent experiments; 10 images acquired for each condition per replicate), with significance calculated by a one-way ANOVA with Tukey’s test. Data are presented as mean ± SEM; * p < 0.05. Representative images are single planes of whole-cell z-stacks; white bars represent the scale bar = 5 μm.

**Figure 2:**
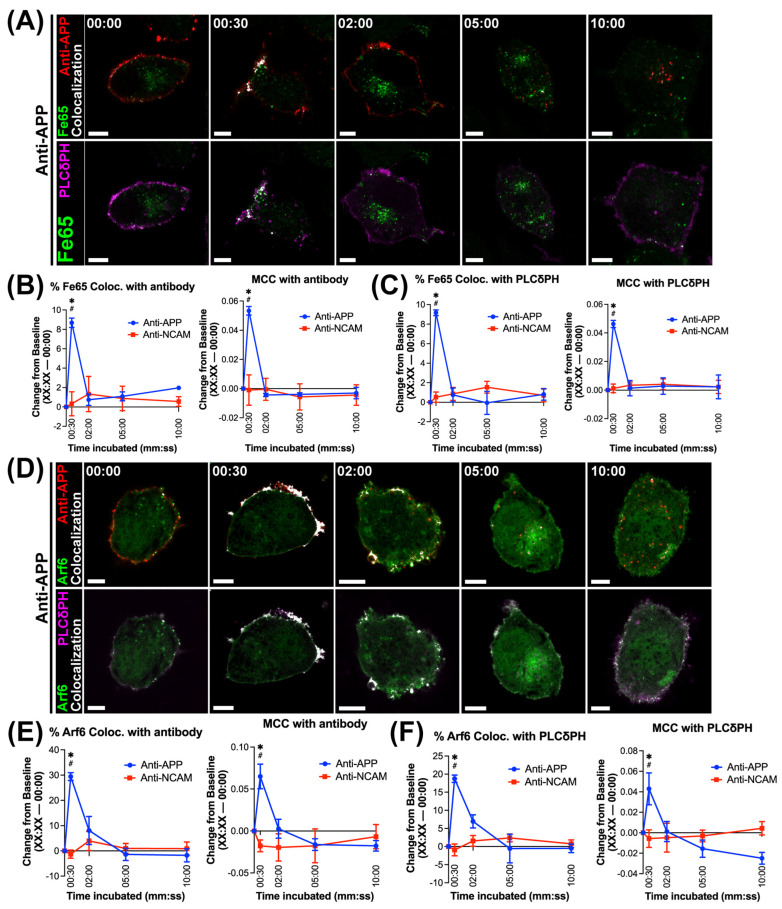
Fe65 and Arf6 are rapidly and transiently recruited to antibody-bound/crosslinked APP and membrane ruffles after 30 s. (**A**) N2a cells transfected with Fe65-EGFP (green), PLCδPH-mRFP (magenta) and APP695. Cells were incubated with fluorescent anti-APP or anti-NCAM antibodies (red) on ice, then immediately fixed on ice as a baseline (00:00) or incubated for 30 s (00:30), 2 min (02:00), 5 min (5:00), and 10 min (10:00), and then fixed and imaged. Colocalization between signals is indicated by white pixels. (**B**,**C**) The quantification of colocalization data (*n* = 3 independent experiments; 15 images acquired for each time point in both conditions per replicate) is represented as the difference between the mean percent colocalized or MCC at each timepoint relative to baseline (XX:XX−00:00). * denotes a significant difference from baseline within group; # denotes a significant difference between groups at the indicated timepoint. (**D**) N2a cells transfected with Arf6-EGFP (green), PLCδPH-mRFP and APP695. The experiment above was repeated with Arf6-EGFP-expressing cells. Colocalization between signals is indicated by white pixels. (**E**,**F**) Quantification of colocalization data (*n* = 3 independent experiments; 15 images acquired for each time point in both conditions per replicate). Data representation and analysis from (**B**,**C**) was repeated for N2a cells expressing Arf6-GFP. * denotes significant difference from baseline within group; # denotes significant difference between groups at the indicated timepoint. Significance was measured by a two-way ANOVA with a Tukey’s test using a single pooled variance. Data are presented as mean ± SEM; */# p < 0.05. Representative images are single planes of whole-cell z-stacks; white bars represent the scale bar = 5 μm.

**Figure 3:**
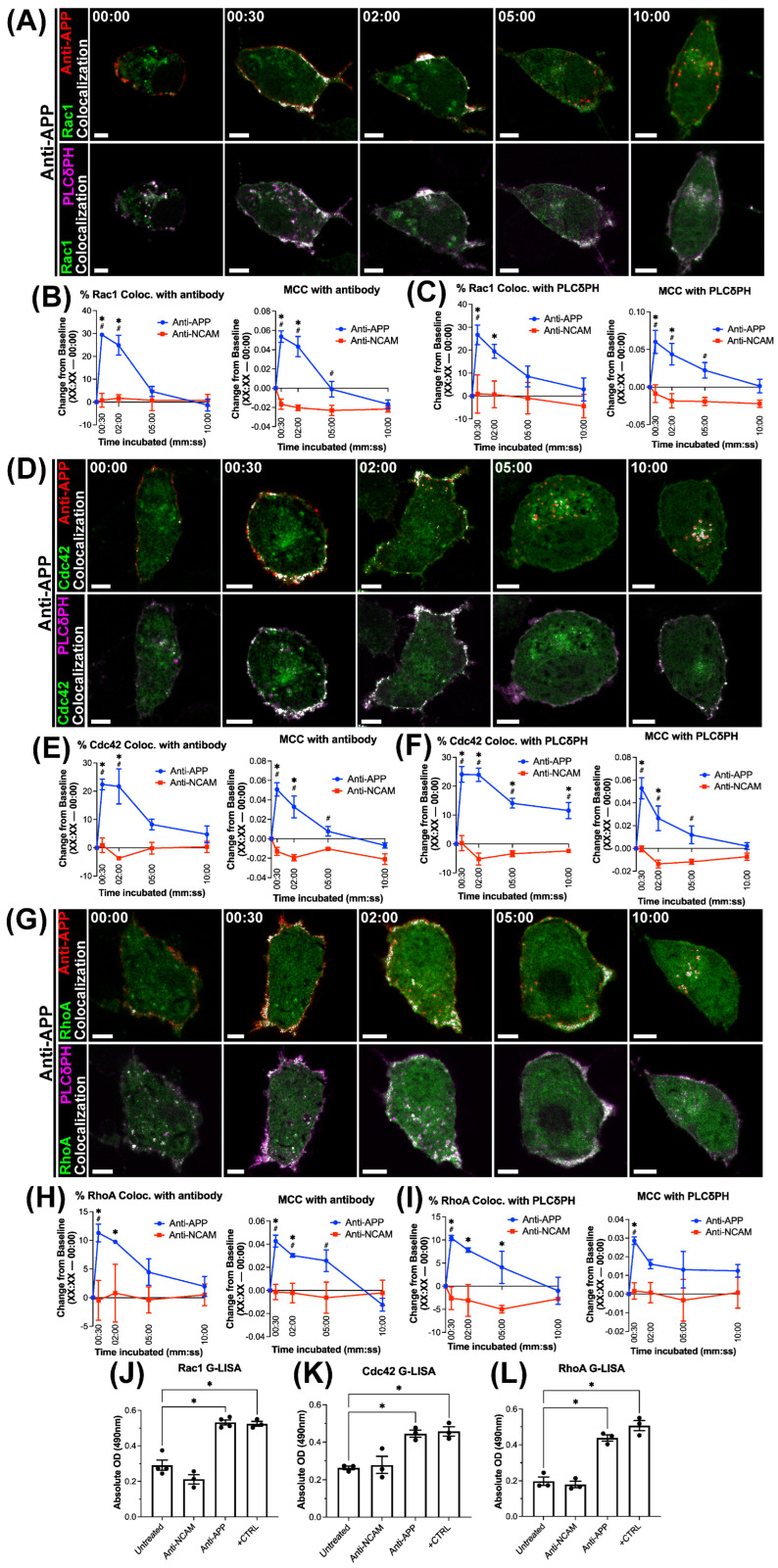
Rac1, Cdc42 and RhoA demonstrate rapid and sustained recruitment to bound/crosslinked APP and membrane ruffles and increased GTP-bound levels. (**A**) N2a cells transfected with Rac1-EGFP (green), PLCδPH-mRFP (magenta) and APP695. Cells were incubated with tagged anti-APP or anti-NCAM antibodies (red) on ice and then were either immediately fixed on ice as a baseline (00:00) or incubated for 30 s (00:30), 2 min (02:00), 5 min (5:00), and 10 min (10:00) at 37 °C prior to fixation. Colocalization between signals is indicated by white pixels. (**B**,**C**) The quantification of colocalization data (*n* = 3 independent experiments; 15 images acquired for each time point in both conditions per replicate) is represented as the difference between the mean percent colocalized or MCC at each timepoint relative to baseline (XX:XX−00:00). N2a cells transfected with (**D**) Cdc42-EGFP or (**G**) RhoA-EGFP, PLCδPH-mRFP and APP695. The experiment from (**A**) was repeated with Cdc42- or RhoA-expressing cells. (**E**,**F**,**H**,**I**) Quantification of colocalization data (*n* = 3 independent experiments; 15 images acquired for each time point in both conditions per replicate). Data representation and analysis from (**B**,**C**) was repeated for N2a cells expressing Cdc42-EGFP. * denotes significant difference from baseline within group; # denotes significant difference between groups at the indicated timepoint. Significance was measured by a two-way ANOVA with a Tukey’s test using a single pooled variance. (**J**) G-LISA assay for Rac1-GTP from lysates of untreated N2a cells or cells incubated on ice with anti-NCAM, anti-APP or EGF (positive control), then incubated for 2 min. Lysates were immediately acquired after and used for Rac1 G-LISA activation assays. To compare the relative amount of GTP-bound Rac1 in each condition, the absolute optical density (OD) was measured from each condition’s lysate loaded into G-LISA assay wells. Experiments were run in technical triplicates across three independent experiments (*n* = 3). Significant differences from untreated samples were calculated by a one-way ANOVA with Tukey’s test. This same experiments and analyses were performed for (**K**) Cdc42-GTP levels and (**L**) RhoA-GTP levels. Data are presented as mean ± SEM; */# p < 0.05. Representative images are single planes of whole-cell z-stacks; white bars represent the scale bar = 5 μm.

**Figure 4:**
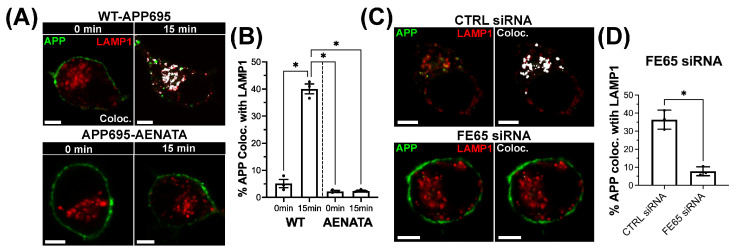
APP mutation of the YENPTY site and siRNA knockdown of FE65 prevents APP macropinocytosis. (**A**) N2a cells transfected with either WT-APP695 (top) or APP-AENATA (bottom) and LAMP1-mCh (red). Cells were incubated with tagged anti-APP antibody (green) on ice for 20 min, then fixed on ice (0 min) or incubated for 15 min then fixed. Colocalization was assessed between bound/crosslinked APP and LAMP1 (white pixels). (**B**) Quantification of the mean % of APP colocalized with LAMP1 from three independent experiments (*n* = 3; 15 images acquired for each condition per replicate) with cells expressing WT-APP or APP-AENATA. Statistical significance was analyzed by a one-way ANOVA with Tukey’s test. (**C**) N2a cells transfected with APP695, LAMP1-mCh, and either non-targeting scramble siRNA (control; CTRL) or FE65 siRNA. Cells were incubated with tagged anti-APP antibody (green) on ice for 20 min, then incubated for 15 min and fixed. The colocalization of APP and LAMP1 was assessed in CTRL and FE65 siRNA-treated cells (white). (**D**) The mean % of APP colocalized with LAMP1 was quantified from three independent experiments (*n* = 3; 10 images acquired for each condition per replicate). Statistical significance between the % colocalized in CTRL and FE65 siRNA-treated cells was analyzed by a two-tailed unpaired *t*-test. Data are presented as mean ± SEM; * p < 0.05. Representative images are single planes of whole-cell z-stacks; white bars represent the scale bar = 5 μm.

**Figure 5:**
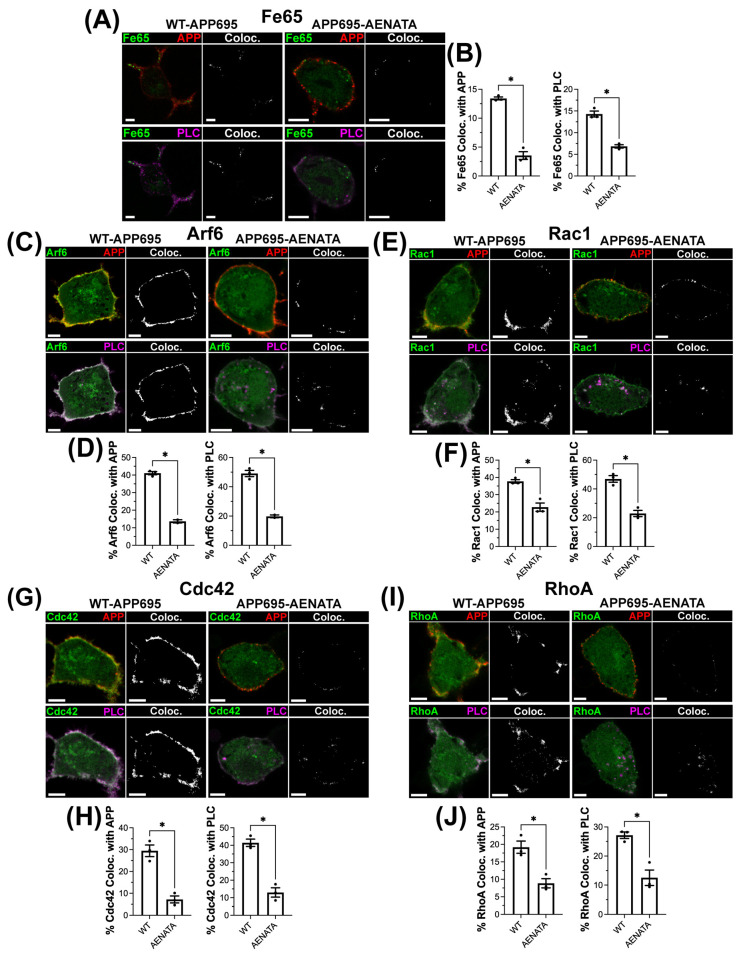
The mutation of the YENPTY site reduces Fe65 and GTPase recruitment to bound/crosslinked APP and membrane ruffles. (**A**) N2a cells transfected with WT-APP or APP-AENATA, Fe65-EGFP (green), and PLCδPH-mRFP (magenta). APP was bound/crosslinked by a tagged APP antibody (red) while on ice, then incubated for 30 s. Colocalization was measured between Fe65 and bound/crosslinked WT-APP or APP-AENATA, as well as between Fe65 and PLCδPH (white pixels). (**B**) The quantification of the mean % of Fe65 colocalized with either bound/crosslinked APP (top) or PLCδPH (bottom) in N2a cells expressing WT-APP or APP-AENATA. The data presented are from three independent experiments (*n* = 3; 15 images acquired for each condition per replicate) with cells expressing WT-APP or APP-AENATA. Significant differences between cells expressing WT-APP or APP-AENATA mutant were analyzed by a two-tailed unpaired *t*-test. This experiment and analysis were repeated with N2a cells expressing (**C**,**D**) Arf6-GFP, (**E**,**F**) Rac1-GFP, (**G**,**H**) Cdc42-GFP, and (**I**,**J**) RhoA-GFP. Data are presented as mean ± SEM; * p < 0.05. Representative images are single planes of whole-cell z-stacks; white bars represent the scale bar = 5 μm.

**Figure 6:**
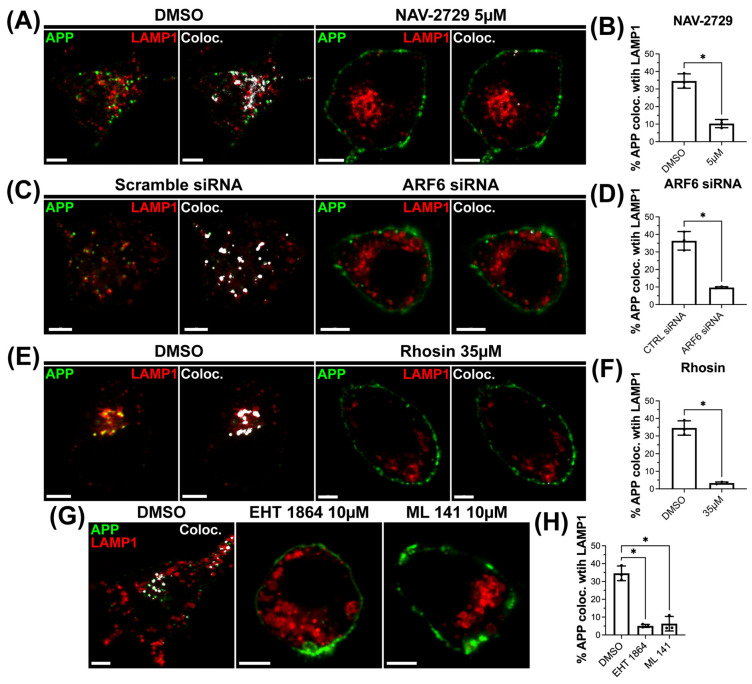
Small GTPase inhibition and siRNA knockdown of Arf6 inhibits the macropinocytosis of APP. (**A**) N2a cells transfected with APP695 and LAMP1-mCh (red) that were treated with DMSO or 5 μM NAV-2729 (Arf6 inhibitor). APP was then bound/crosslinked by fluorescent tagged APP antibodies and imaged after a 15 min incubation. Colocalization was assessed between crosslinked APP and LAMP1 (white pixels). (**B**) Quantification of the mean % of APP colocalized with LAMP1 (*n* = 3 independent experiments; 10 images per replicate), with significance calculated by a two-tailed unpaired *t*-test. (**C**) N2a cells transfected with APP695, LAMP1-mCh and either non-targeting scramble siRNA (control; CTRL) or ARF6 siRNA. Following transfection and differentiation, APP was bound/crosslinked by fluorescent-tagged APP antibodies and imaged after 15 min incubation. Colocalization was assessed between and LAMP1 (white pixels). (**D**) Quantification of the mean % of APP colocalized with LAMP1 (*n* = 3 independent experiments; 10 images per replicate), with significance calculated by a two-tailed unpaired *t*-test. (**E**) N2a cells transfected with APP695 and LAMP1-mCh (red) that were treated with DMSO or 35 μM Rhosin (RhoA inhibitor). APP was then bound/crosslinked by fluorescent-tagged APP antibodies and imaged after a 15 min incubation. Colocalization was assessed between crosslinked APP and LAMP1 (white pixels). (**F**) Quantification of the mean % of APP colocalized with LAMP1 (*n* = 3 independent experiments; 10 images per replicate), with significance calculated by a two-tailed unpaired *t*-test. (**G**) N2a cells transfected with APP695 and LAMP1-mCh (red) that were treated with DMSO, 10 μM EHT 1864 (Rac1 inhibitor), or 10 μM ML 141 (Cdc42 inhibitor). APP was then bound/crosslinked by fluorescent-tagged APP antibodies and imaged after 15 min incubation. Colocalization was assessed between crosslinked APP and LAMP1 (white pixels). (**H**) Quantification of the mean % of APP colocalized with LAMP1 (*n* = 3 independent experiments; 10 images per replicate), with significance calculated by a one-way ANOVA with Tukey’s test. Data are presented as mean ± SEM; * p < 0.05. Representative images are single planes of whole-cell z-stacks; white bars represent the scale bar = 5 μm.

**Figure 7:**
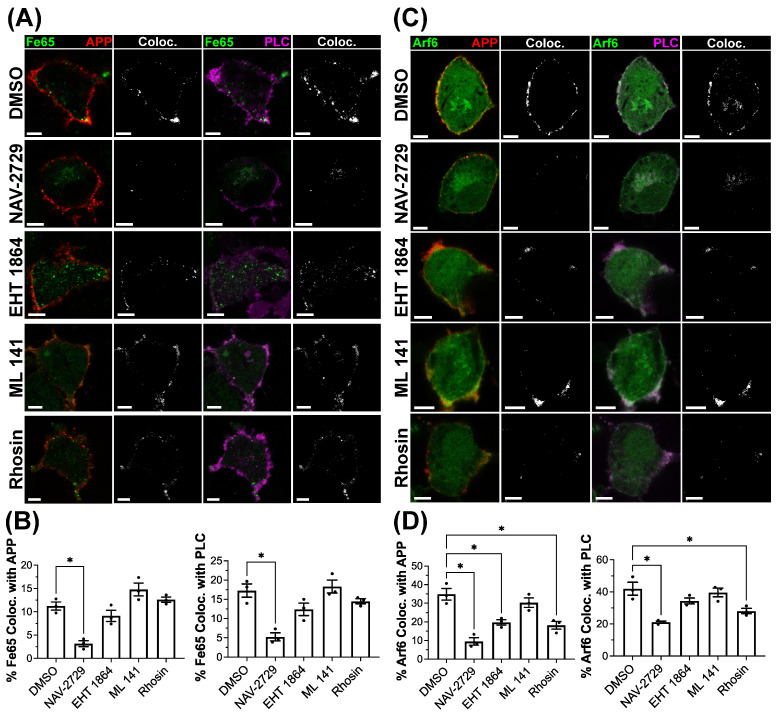
Effects of GTPase inhibition on the recruitment of Fe65 and Arf6 to bound/crosslinked APP and membrane ruffles at 30 s. (**A**) N2a cells transfected with Fe65-EGFP (green), PLCδPH-mRFP (magenta) and APP695. Cells were treated with 0.1% DMSO, 5 μM NAV-2729, 10 μM EHT 1864, 10 μM ML 141, and 35 μM Rhosin. After treatment, cells were incubated with tagged anti-APP antibodies (red) on ice and then fixed following an incubation for 30 s. Colocalization was assessed between Fe65 and antibody bound/crosslinked APP or PLCδPH (white pixels). (**B**) Quantification of the mean % of Fe65 colocalized with APP (left) or PLCδPH (right) from three independent experiments (*n* = 3; 15 images acquired for each condition per replicate). (**C**) N2a cells transfected with Arf6-EGFP (green), PLCδPH-mRFP and APP695. Cells were treated with 0.1% DMSO, 5 μM NAV-2729, 10 μM EHT 1864, 10 μM ML 141, and 35 μM Rhosin. After treatment, cells were incubated with tagged anti-APP antibodies (red) on ice and then fixed following a 30 s incubation. Colocalization was assessed between Arf6 and bound/crosslinked APP or PLCδPH (white pixels). (**D**) Quantification of the mean % of Arf6 colocalized with APP (left) or PLCδPH (right) from three independent experiments (*n* = 3; 15 images acquired for each condition per replicate). Significant changes in the percentage colocalized between treatments were assessed by a one-way ANOVA with Tukey’s test. Data are presented as mean ± SEM; * p < 0.05. Representative images are single planes of whole-cell z-stacks; white bars represent the scale bar = 5 μm.

**Figure 8:**
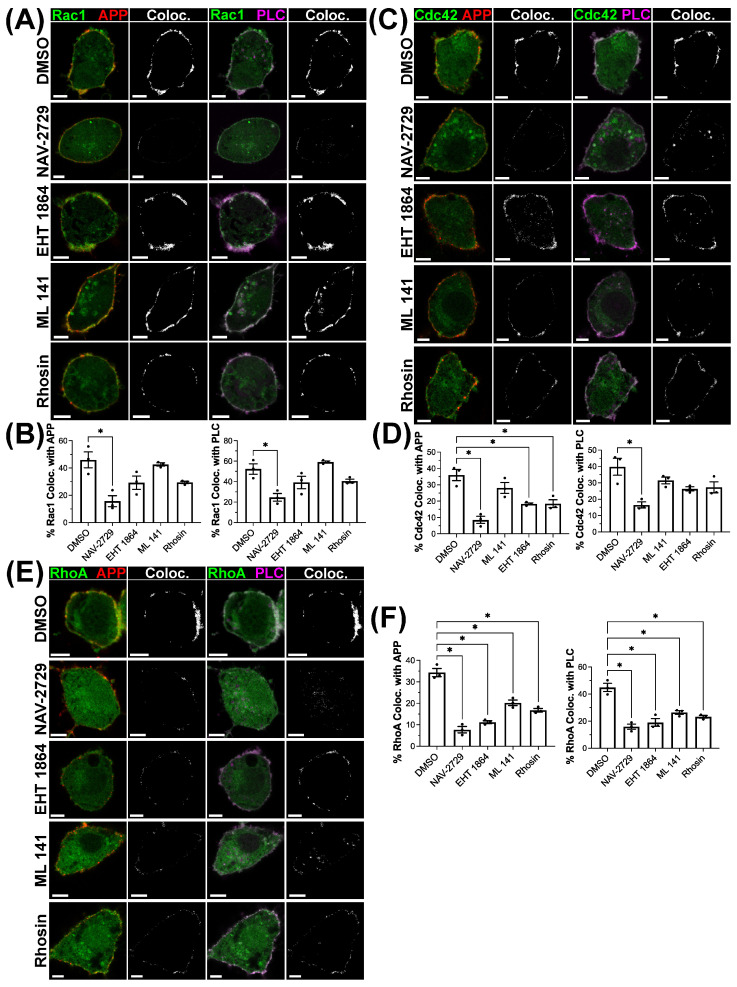
Effects of GTPase inhibition on the recruitment of the Rho GTPases Rac1, Cdc42 and RhoA to bound/crosslinked APP and membrane ruffles at 30 s. (**A**) N2a cells transfected with Rac1-EGFP (green), PLCδPH-mRFP (magenta) and APP695. Cells were treated with 0.1% DMSO, 5 μM NAV-2729, 10 μM EHT 1864, 10 μM ML 141, and 35 μM Rhosin. After treatment, cells were incubated with tagged anti-APP antibodies (red) on ice and then fixed following a 30 s incubation. Colocalization was assessed between Rac1 and bound/crosslinked APP or PLCδPH (white pixels). (**B**) Quantification of the mean % of Rac1 colocalized with APP (left) and PLCδPH (right) from three independent experiments (*n* = 3; 15 images acquired for each condition per replicate). N2a cells transfected with (**C**) Cdc42-EGFP or (**E**) RhoA-EGFP, PLCδPH-mRFP and APP695. Cells were treated as described above, then incubated with tagged anti-APP antibodies and fixed following a 30 s incubation. Colocalization with APP or PLCδPH was measured (white pixels). (**D**,**F**) Quantification of the mean % of Cdc42 or RhoA colocalized with APP (left) and PLCδPH (right) from three independent experiments (*n* = 3; 15 images acquired for each condition per replicate). Significant changes in the percentage colocalized between treatments were assessed by a one-way ANOVA with Tukey’s test. Data are presented as mean ± SEM; * p < 0.05. Representative images are single planes of whole-cell z-stacks; white bars represent the scale bar = 5 μm.

**Table 1:** Forward and reverse primers used for the qPCR quantification of FE65 and ARF6 knockdown.

Gene	Forward Primer ^1^	Reverse Primer ^1^
Rpl13α	GCTGTGAGGGCATCAACATT	TTGGTGTTCATCCGCTTTCG
Apbb1 (FE65) Arf6	CACCGAGACCAGAACCGAAA TCTGGCGGCATTACTACACC	AGGTAGCCCTGGAGGAGAAG GAGGGCTGCACATACCAGTT

^1^ Primers are written from 5′ to 3′.

## Data Availability

The raw data supporting the conclusions of this article will be made available by the authors on request. Raw Western blot images are available in the Supplementary Materials. At the time of submission, there are technical and time limitations that prevent us from making the raw microscopy data publicly available due to the size of the data set. Any data requested will be provided by the authors (J.M.K. or S.H.P.).

## Supplementary Materials

The following supporting information can be downloaded at: https://www.mdpi.com/article/10.3390/cells15151366/s1.

## Abbreviations

The following abbreviations are used in this manuscript:

βCTF	β-C-terminal fragment
APP	Amyloid Precursor Protein
Aβ	Amyloid-beta
Arf6	ADP-ribosylation factor 6
Cdc42	Cell division control protein 42 homolog
CME	Clathrin-mediated endocytosis
EIPA	5-(N-ethyl-N-isopropyl) amiloride
GFLD	Growth factor-like domain
LAMP-1	Lysosomal associated membrane protein 1
mChFP	Monomeric cherry fluorescent protein
NCAM	Neural cell adhesion molecule
NGF	Nerve growth factor
PLC	Phospholipase C
PI	Phosphatidylinositol
PH	Pleckstrin homology domain
Rac1	Ras-related C3 botulinum toxin substrate 1
RhoA	Ras homolog family member A
