# Supplementary material for: Macropinocytosis of Amyloid Precursor Protein Is Regulated by the Recruitment and Activity of Fe65, Arf6 and Rho GTPases
Source: Cells. 2026 Jul 29;15(15):1366. doi: 10.3390/cells15151366 (PMC13464792; doi:10.3390/cells15151366)
Supplement: Supplementary file 1 [file cells-15-01366-s001.zip › cells-4385457-supplementary.pdf]

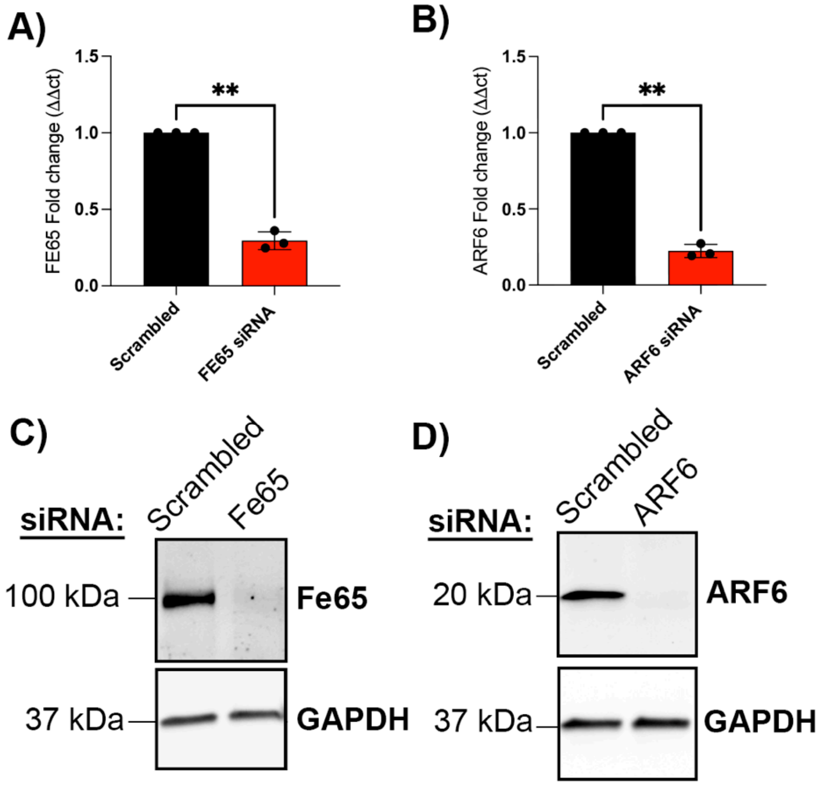

Figure S1. Quantification of Fe65 and Arf6 knockdown by qPCR and Western blot.
N2a cells were transfected with a scrambled non-targeting (control) siRNA and either FE65 siRNA or ARF6 siRNA. Total RNA was isolated from transfected cells, cDNA was generated from RNA isolates, and fold change (ΔΔCt) in expression was measured by qPCR relative to expression levels of RPL13α. Significant difference in fold change (ΔΔCt) between scrambled siRNA and **A)** FE65 siRNA or **B)** ARF6 siRNA was calculated by a two-way unpaired t-test. The same experimental set up as above was performed, followed by total protein extraction for Western blotting. Protein extraction isolate concentrations were determined by BCA protein assay and 20µg of total protein for each isolate was separated using SDS-PAGE then transferred onto a PVDF membrane. Following blocking and incubation with Fe65, Arf6 or GAPDH (control) antibodies, membranes were incubated with HRP-conjugated secondary antibodies then imaged to visualize protein bands. **C)** FE65 siRNA treatment resulted in disappearance of the 100 kDa band corresponding to Fe65. **D)** ARF6 siRNA treatment resulted in disappearance of the 20 kDa band corresponding to Arf6. *Data is presented as mean ± SEM; **p<0.01.*

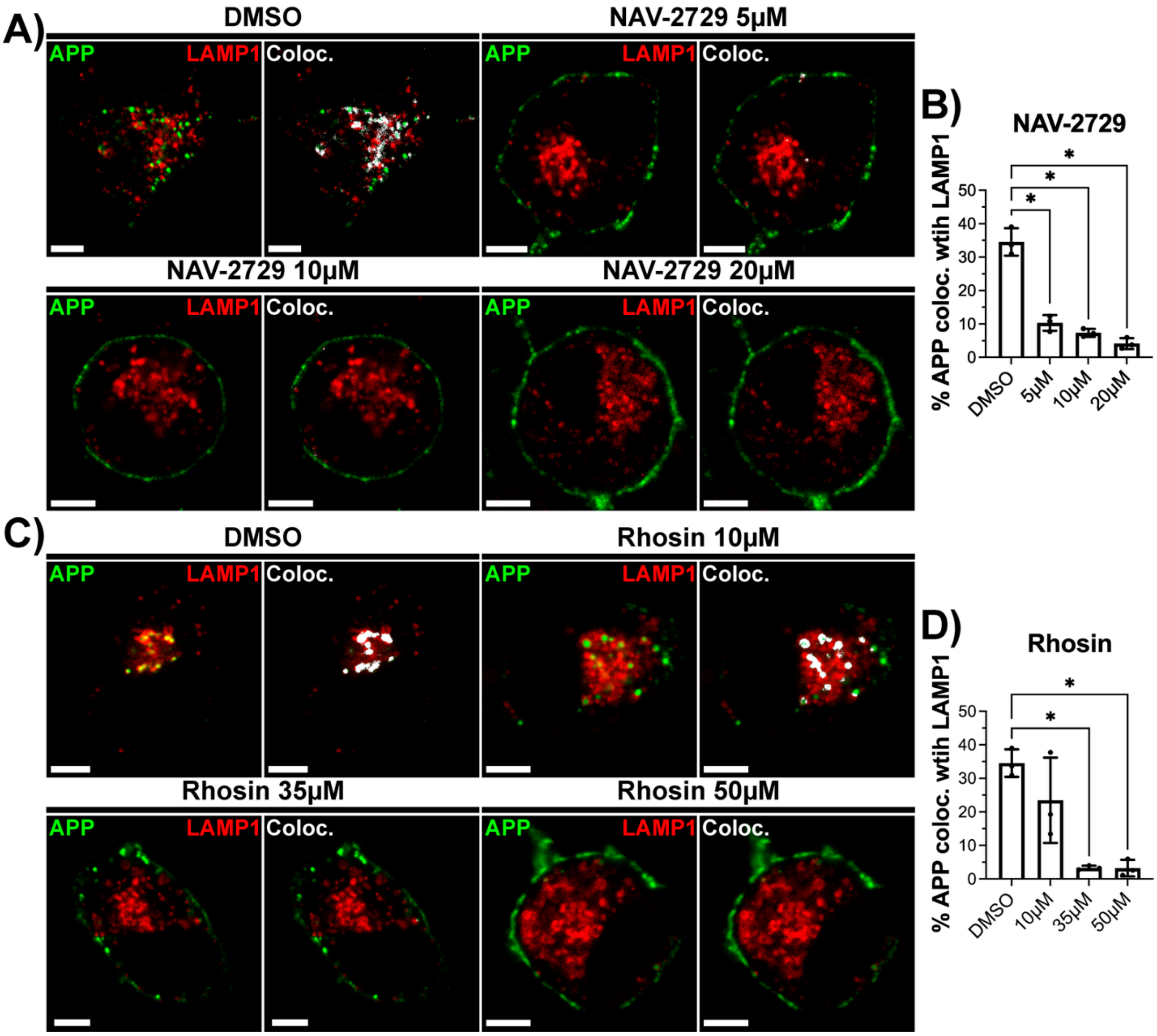

Figure S2. Titration of NAV-2729 and Rhosin dose to inhibit the internalization of APP by macropinocytosis.

A) N2a cells transfected with APP695 and LAMP1-mCh (red) that were treated with DMSO, 5µM, 10µM, or 20µM NAV-2729 (Arf6 inhibitor). APP was then bound/crosslinked by fluorescent tagged APP antibodies and imaged after 15 minute incubation. Colocalization was assessed between crosslinked APP and LAMP1 (white pixels). **B)** Quantification of the mean % of APP colocalized with LAMP1 (n=3 independent experiments; 10 images per replicate), with significance calculated by a one-way ANOVA with Tukey's test. **C)** N2a cells transfected with APP695 and LAMP1-mCh (red) that were treated with DMSO, 10µM, 25µM, or 50µM Rhosin (RhoA inhibitor). APP was then bound/crosslinked by fluorescent tagged APP antibodies and imaged after 15 minute incubation. Colocalization was assessed between crosslinked APP and

LAMP1 (white pixels). **D)** Quantification of the mean % of APP colocalized with LAMP1 (n=3 independent experiments; 10 images per replicate), with significance calculated by a one-way ANOVA with Tukey's test. *Data is presented as mean ± SEM; *p<0.05. Representative images are single planes of whole-cell z-stacks; scale bar = 5µm*

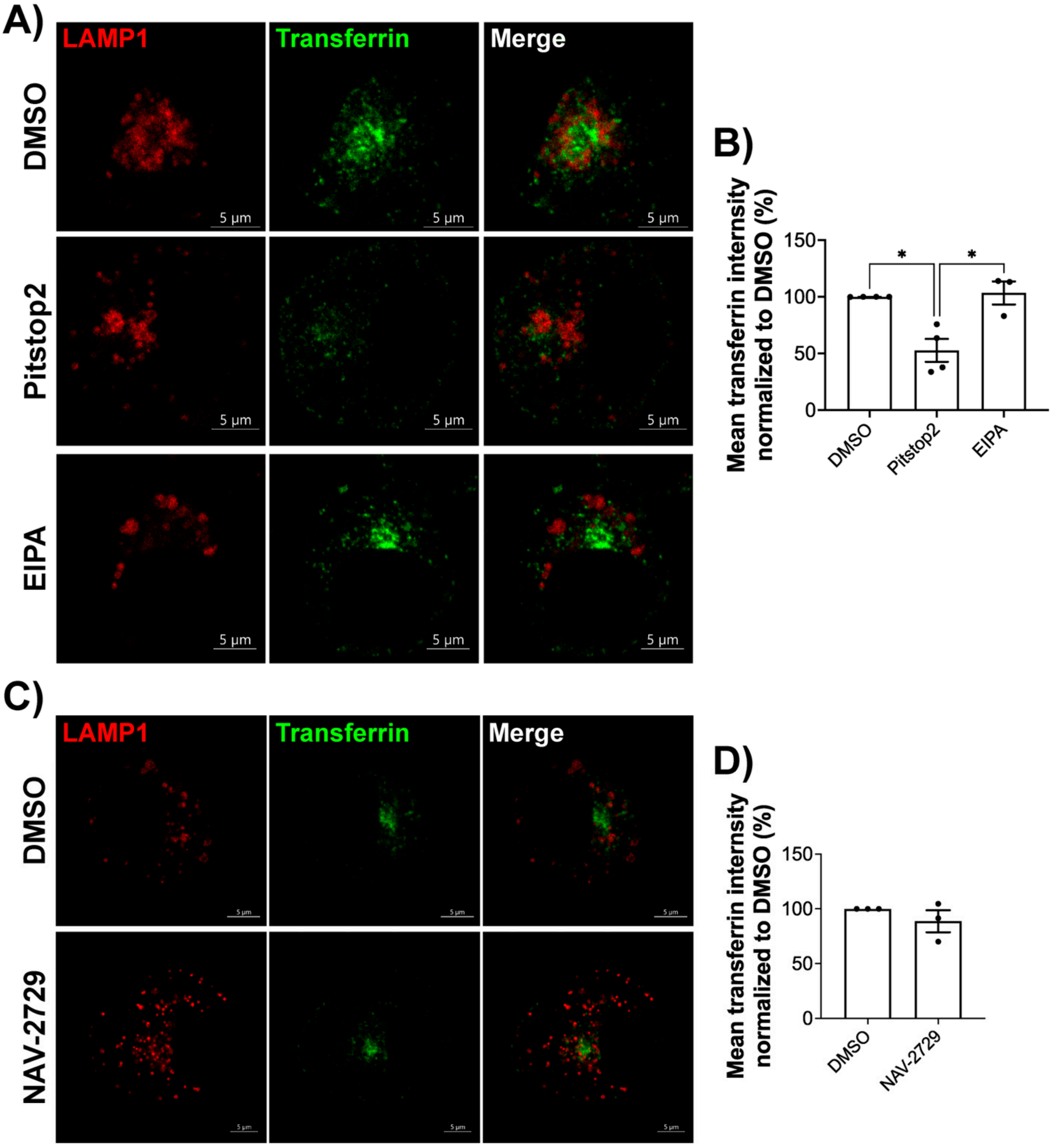

Figure S3. Pitstop2 inhibition of CME reduces the amount of transferrin internalized in N2a cells and NAV-2729 inhibition has no effect on transferrin internalization.

A) N2a cells transfected with LAMP1-mCh (red) that were treated with DMSO (vehicle control), 20µM Pitstop2 (CME inhibitor), or 10µM EIPA (macropinocytosis inhibitor). Transfected cells were incubated with Alexa488-tagged transferrin on ice for 20 minutes then fixed and imaged after a 15 minute incubation. Imaging settings were kept consistent across images taken and the mean

pixel intensity of transferring was measured in each image across all conditions. **B)** Quantification of changes in mean pixel intensity of transferrin signal within N2a cells across treatment conditions (n=3; 10 images per replicate), with significance calculated by a one-way ANOVA with Tukey's test. Mean pixel intensity of transferrin signal was normalized to DMSO vehicle control. Significantly reduction in mean pixel intensity of transferrin signal was observed in response to Pitstop2 treatment indicating inhibition of CME. **C)** The same experimental set up was used to assess the effects of the inhibitor NAV-2729 on transferrin uptake. Prior to the experiment cells were treated with 5µM of NAV-2729, as described in the main text of this study. **B)** Quantification of changes in mean pixel intensity of transferrin signal within N2a cells across treatment conditions (n=3 independent experiments; 10 images per replicate), with significance calculated by a two-tailed unpaired t-test. Mean pixel intensity of transferrin signal was normalized to DMSO vehicle control. *Data is presented as mean ± SEM; * $p<0.05$. Representative images are single planes of whole-cell z-stacks; scale bar = 5µm.*

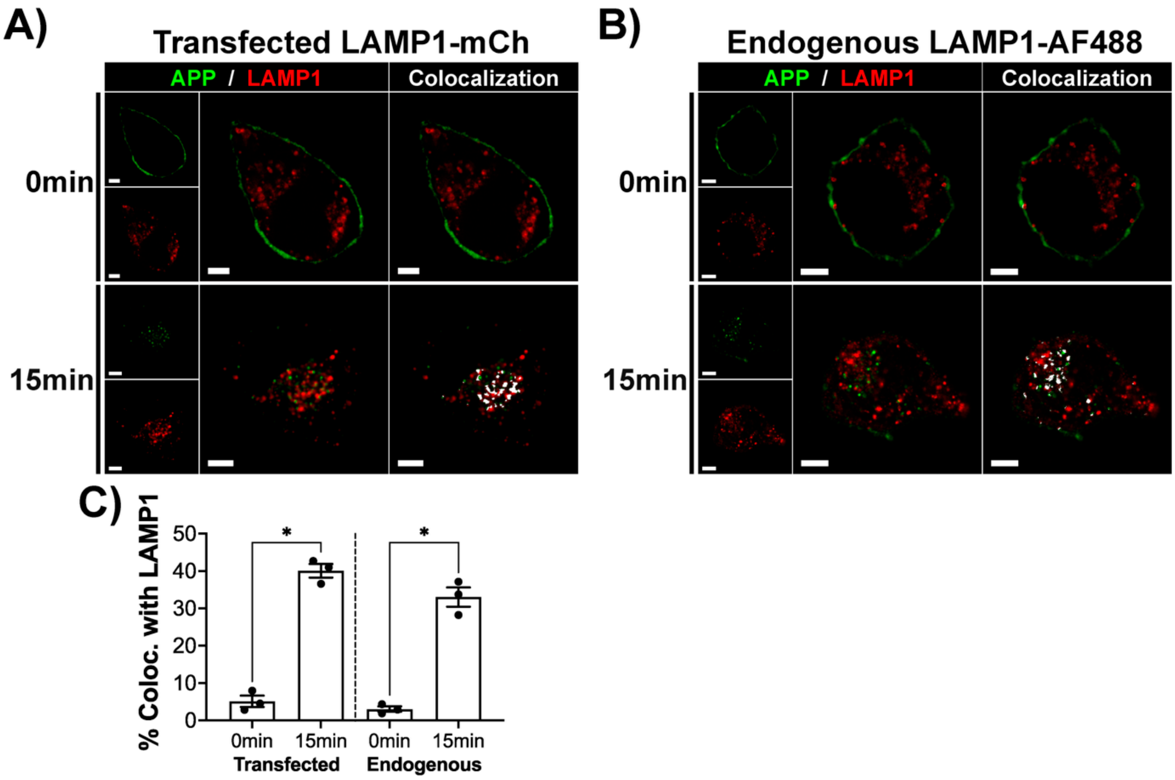

Figure S4. Antibody bound/crosslinked APP demonstrates similar internalization to LAM1 labeled compartments in LAMP1 overexpressing cells and endogenously stained cells. **A)** N2a cells transfected with APP695 and LAMP1-mCh or **B)** APP695 alone and LAMP1 was immunolabeled following fixation. Cells were incubated with fluorescent-tagged anti-APP antibodies (red) on ice, then immediately fixed on ice (0min) or incubated for 15 minutes then fixed. Colocalization between antibody bound/crosslinked APP and overexpressed or endogenous LAMP1 signal is indicated by white pixels. **C)** Quantification of mean % of APP colocalized with transfected or endogenous LAMP1 (n=3 independent experiments; 10 images per replicate). Significance was calculated by a one-way ANOVA with Tukey's test. *Data is presented as mean ± SEM; * $p<0.05$. Representative images are single planes of whole-cell z-stacks; scale bar = 5µm.*

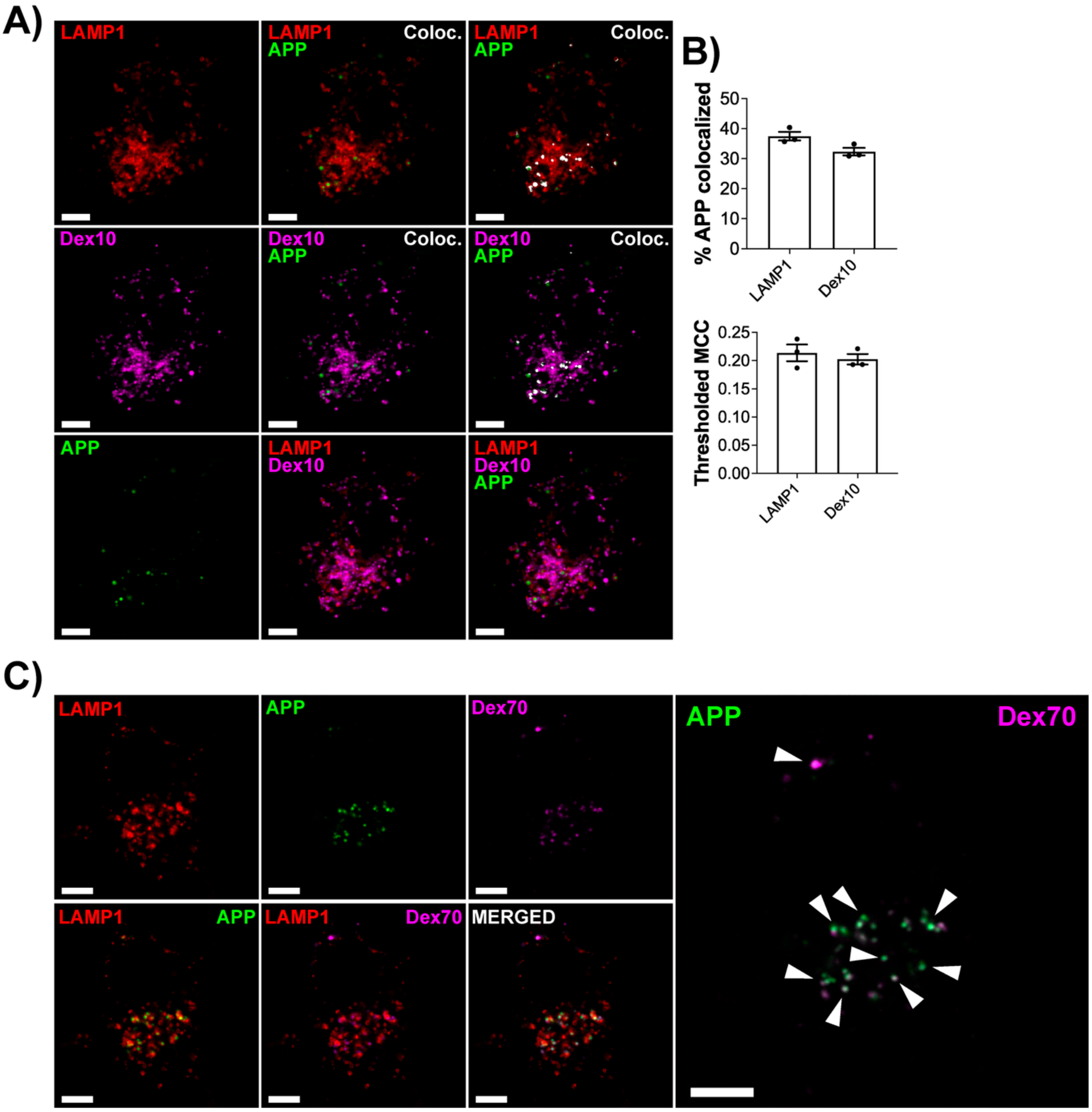

Figure S5. Antibody bound/crosslinked APP internalizes to Dex10 loaded compartments and co-uptakes with Dex70.

A) N2a cells transfected with with APP695 and LAMP1-CFP (red). Prior to antibody mediated binding/crosslinking of APP, cells were pulse loaded with 1mg/mL of Dex10-TMR for 6 hours then chased for 16 hours prior to the experiment. Dex10 loaded cells were incubated with fluorescent-tagged anti-APP antibodies (red) on ice, then incubated for 15 minutes to allow to internalize prior to fixation. Colocalization between APP and LAMP1 or Dex10 signal is indicated by white pixels. **B)** Quantification of mean % of APP colocalized with LAMP1 or

Dex10 (n=3 independent experiments; 10 images per replicate). Significance was calculated by a two-tailed unpaired t-test. **C)** APP695 and LAMP1-CFP transduced N2a cells underwent antibody mediated APP binding/crosslinking on ice as previously described. Before removal from ice, 1mg/mL of Dex70-TMR was added and cells were then allowed to incubate to allow for APP and Dex70 internalization. White arrows depict puncta where APP and Dex70 signal overlap. *Data is presented as mean ± SEM; * $p<0.05$. Representative images are single planes of whole-cell z-stacks; scale bar = 5µm.*

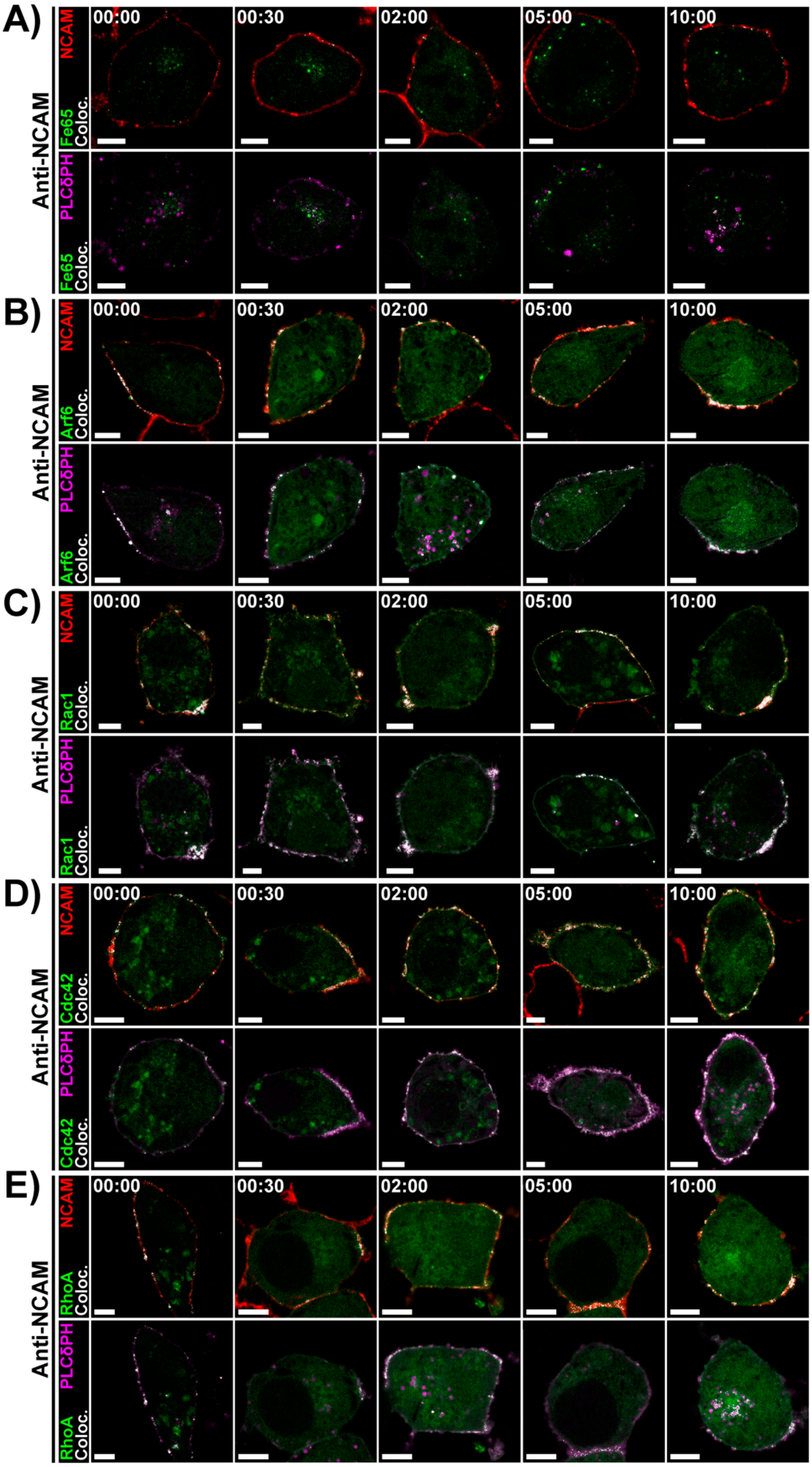
A)
Anti-NCAM
NCAM
Fe65
Coloc.
PLCδPH
Fe65
Coloc.
00:00
00:30
02:00
05:00
10:00
B)
Anti-NCAM
NCAM
Arf6
Coloc.
PLCδPH
Arf6
Coloc.
00:00
00:30
02:00
05:00
10:00
C)
Anti-NCAM
NCAM
Rac1
Coloc.
PLCδPH
Rac1
Coloc.
00:00
00:30
02:00
05:00
10:00
D)
Anti-NCAM
NCAM
Cdc42
Coloc.
PLCδPH
Cdc42
Coloc.
00:00
00:30
02:00
05:00
10:00
E)
Anti-NCAM
NCAM
RhoA
Coloc.
PLCδPH
RhoA
Coloc.
00:00
00:30
02:00
05:00
10:00

Figure S6. Antibody-mediated binding/crosslinking of NCAM negative control for regulatory protein recruitment.

A) N2a cells transfected with Fe65-EGFP (green), PLCδPH-mRFP (magenta) and APP695. Cells were incubated with fluorescent-tagged anti-NCAM antibodies (red) on ice, then immediately fixed on ice as a baseline (00:00) or incubated for 30 seconds (00:30), 2 minutes (02:00), 5 minutes (5:00), and 10 minutes (10:00) and then fixed. Colocalization between Fe65-EGFP and anti-NCAM or PLCδPH-mRFP signal is indicated by white pixels. This experiment was repeated with cells expressing **B)** Arf6-GFP, **C)** Rac1-GFP, **D)** Cdc42-GFP and **E)** RhoA-GFP. *Representative images are single planes of whole-cell z-stacks; scale bar = 5µm.*

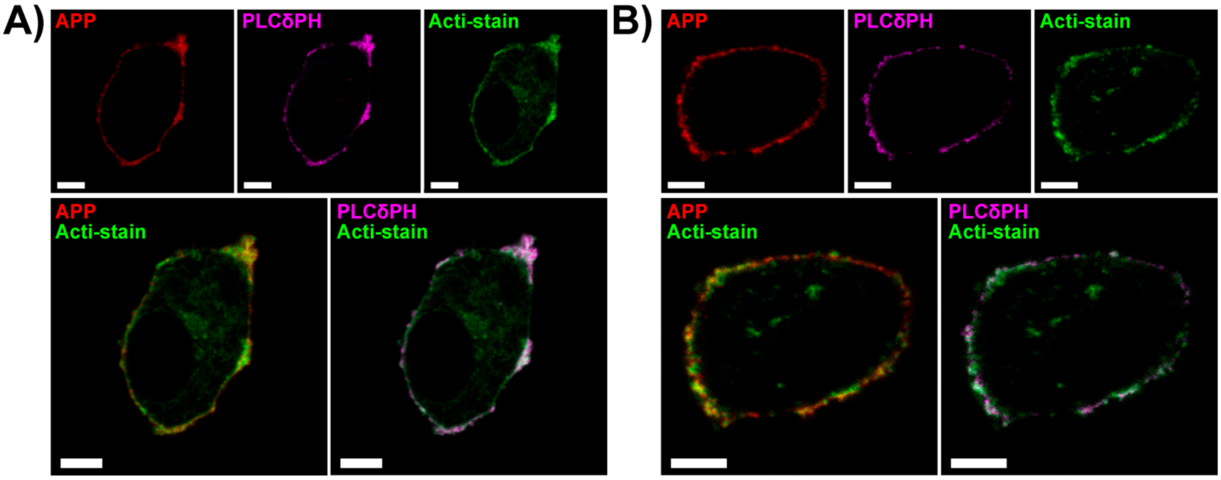

Figure S7. Antibody bound/crosslinked APP and PLCδPH localize at the plasma membrane with phalloidin-labeled F-actin after incubation for 30 seconds and 2 minutes.

A) N2a cells transfected with APP695 and PLCδPH-mRFP (magenta) underwent antibody mediated APP binding/crosslinking (red) on ice, then incubated for 30 seconds. After fixation F-actin was labeled using Acti-stain 555 (green). Bright accumulations of each signal can be appreciated overlapping at the plasma membrane. **B)** This same experiment was repeated with cells incubated for 2 minutes following APP binding/crosslinking. All three signals are observed at this time point overlapping at the plasma membrane. *Representative images are single planes of whole-cell z-stacks; scale bar = 5µm.*

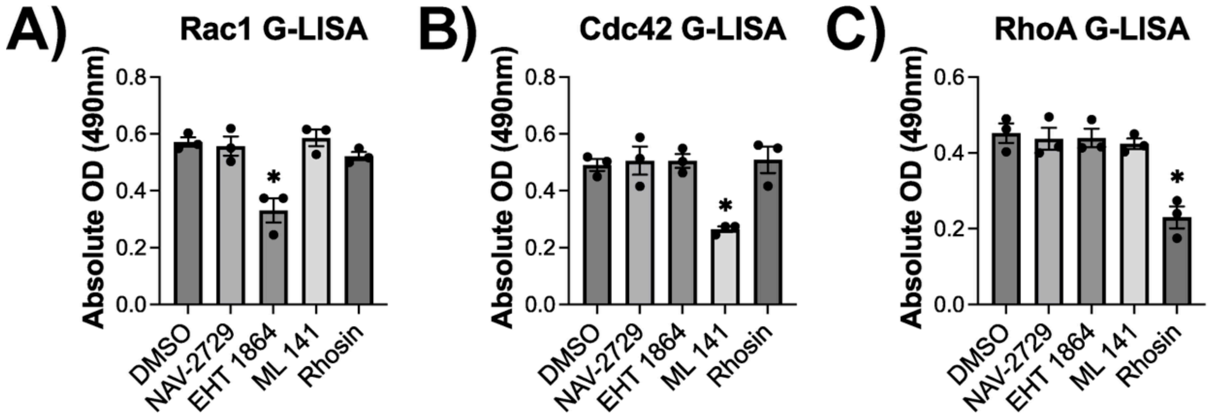

Figure S8. Small molecules used to inhibit specific small GTPase have no effect on active GTP-bound levels of off-target GTPases.

A) G-LISA assay for Rac1-GTP from N2a cells treated with 0.1% DMSO, 5µM NAV-2729, 10µM EHT 1864, 10µM ML 141, and 35µM Rhosin as used in the experiments above. After treatment, lysates were immediately collected and used for Rac1 G-LISA activation assays. To compare the relative amount of active GTP-bound Rac1 in each treatment condition, the absolute optical density (OD) was measured from each condition's lysate loaded into G-LISA assay wells. Three different lysate samples were collected for each condition from different biological samples (n=3), and the G-LISA assay was performed in technical triplicates. Significant differences from untreated samples were calculated by a one-way ANOVA with Tukey's test. This same experiment and analysis was performed for **B)** Cdc42-GTP levels, and **C)** RhoA-GTP levels. *Data is presented as mean ± SEM; *p<0.05.*
